# Optical High Content Nanoscopy of Epigenetic Marks Decodes Phenotypic Divergence in Stem Cells

**DOI:** 10.1038/srep39406

**Published:** 2017-01-04

**Authors:** Joseph J. Kim, Neal K. Bennett, Mitchel S. Devita, Sanjay Chahar, Satish Viswanath, Eunjee A. Lee, Giyoung Jung, Paul P. Shao, Erin P. Childers, Shichong Liu, Anthony Kulesa, Benjamin A. Garcia, Matthew L. Becker, Nathaniel S. Hwang, Anant Madabhushi, Michael P. Verzi, Prabhas V. Moghe

**Affiliations:** 1 Department of Biomedical Engineering, Rutgers University, Piscataway, New Jersey, USA; 2 Cardiovascular Institute, Stanford University School of Medicine, Stanford, California, USA; 3 Department of Cell Biology and Neuroscience, Rutgers University, Piscataway, New Jersey, USA; 4 Department of Genetics, Rutgers University, Piscataway, New Jersey, USA; 5 Department of Biomedical Engineering, Case Western Reserve University, Cleveland, Ohio, USA; 6 School of Chemical and Biological Engineering, Seoul National University, Seoul, Republic of Korea; 7 Division of Heath Sciences and Technology, Massachusetts Institute of Technology, Cambridge, MA, USA; 8 Department of Molecular Biology, Princeton University, Princeton, New Jersey, USA; 9 Department of Polymer Science, University of Akron, Akron, Ohio, USA; 10 Epigenetics Program, Department of Biochemistry and Biophysics, Perelman School of Medicine, University of Pennsylvania, Philadelphia, Pennsylvania, USA; 11 Department of Biological Engineering, Massachusetts Institute of Technology, Cambridge, Massachusetts, USA; 12 Department of Chemical and Biochemical Engineering, Rutgers University, Piscataway, New Jersey, USA

## Abstract

While distinct stem cell phenotypes follow global changes in chromatin marks, single-cell chromatin technologies are unable to resolve or predict stem cell fates. We propose the first such use of optical high content nanoscopy of histone epigenetic marks (epi-marks) in stem cells to classify emergent cell states. By combining nanoscopy with epi-mark textural image informatics, we developed a novel approach, termed EDICTS (*Epi-mark Descriptor Imaging of Cell Transitional States*), to discern chromatin organizational changes, demarcate lineage gradations across a range of stem cell types and robustly track lineage restriction kinetics. We demonstrate the utility of EDICTS by predicting the lineage progression of stem cells cultured on biomaterial substrates with graded nanotopographies and mechanical stiffness, thus parsing the role of specific biophysical cues as sensitive epigenetic drivers. We also demonstrate the unique power of EDICTS to resolve cellular states based on epi-marks that cannot be detected via mass spectrometry based methods for quantifying the abundance of histone post-translational modifications. Overall, EDICTS represents a powerful new methodology to predict single cell lineage decisions by integrating high content super-resolution nanoscopy and imaging informatics of the nuclear organization of epi-marks.

A major challenge in regenerative medicine is the robust conversion of heterogeneous stem cell populations into homogenous lineage-restricted cells, and their sustained retention as differentiated phenotypes. Approaches to track how individual stem cells navigate the process of differentiation and how populations adopt the emergent heterogeneity are central to addressing this challenge. Traditional techniques to detect the lineage conversion of stem cells include proteomic^1^,^2^,^3^, genomic^4^,^5^,^6^, and transcriptomic^7^,^8^,^9^,^10^ analyses, which are highly informative but are unable to comprehensively capture the early emergence of differentiation at the single cell level. Consequently, population-level measures of differentiation are frequently aggregated and lack individual-cell resolution. Further, none of the existing techniques capture spatial changes in genome organization, which have been known to accompany cell state changes.

We propose a new technique called **E**pi-mark **D**escriptor **I**maging for **C**ell **T**ransitional **S**tates (EDICTS). EDICTS is based on the hypothesis that the phenotypic divergence in stem cells can be linked to the perturbation of gene organizational domains within the nucleus, which can be detected and parametrized to the extent that the derived parameters can forecast a specific lineage choice. Cellular identity is established via the spatiotemporal expression of distinct phenotype-specific gene programs, which are largely influenced by chromatin structural dynamics that regulate the accessibility of genes to transcriptional machinery. Thus it is expected that modifications to chromatin architecture may precede detectable changes in gene expression that ultimately drive cellular differentiation. Identification of these predictive chromatin structural properties would offer a new opportunity to recognize cellular commitment, prior to the ability to detect downstream distinguishable differences in gene and protein expression using commonly employed differentiation assays. Chromatin-mediated control of gene expression has become increasingly appreciated since the proposition of the “histone code hypothesis”, which posits that combinatorial chemical modifications of histone tails convey regulatory information for transcriptional processes^11^. The subsequent breakthrough in sequencing technologies has spawned the field of epigenomics, which investigates the distribution of these epigenetic marks at the genome scale. Studies of particular epi-marks have revealed underappreciated mechanisms of cellular differentiation^12^,^13^, reprogramming back to pluripotency^14^,^15^, and lineage conversion^16^,^17^,^18^.

Current approaches to interrogating the epigenome, using mass spectrometry and chromatin immunoprecipitation, report on population-level changes, but lack the spatial fidelity to resolve the epigenetic activity driving intranuclear reorganization at the single-cell level^19^,^20^. Moreover, despite their prominent role in navigating the lineage commitment process, epi-marks have not been analyzed to explicitly predict cell differentiation events. We propose that the spatial organization and distribution of specific epi-marks can serve as predictive markers to probe a cell’s response to its environment^21^. Characterizing the 3D organization of epi-marks in the nucleus requires imaging approaches with sufficient spatial resolution. Recent advances in super-resolution microscopy using Stimulated Emission Depletion (STED)^22^,^23^ resolve below Abbe’s diffraction barrier^24^,^25^,^26^.

We advance the EDICTS paradigm based on the combination of super-resolution imaging of single cell organizational features of epi-marks and the underlying bioimage informatics^27^,^28^ (Fig. 1). Dual features of epigenome regulation, namely, the expression of histone post-translational modifications (PTMs) and higher-order chromatin architecture, were captured using time-gated stimulated emission depletion (G-STED) super-resolution imaging and high content data analytics to quantify the intranuclear organizational patterns of epi-marks at the scale of small groups of individual nucleosomes. Of the series of varied image feature filters screened, the most sensitive organizational patterns that accurately classified images of epi-marks were found to be a family of texture features composed of over 100 unique quantitative descriptors^29^. These quantitative descriptors are based on algorithms of Haralick type texture features, which are computed from grey-level co-occurrence matrices (GLCMs) sensitive to the organizational distribution of neighboring pixel values (Supplementary Table 1). We applied high content nanoscopy and texture analysis of nucleus-wide organization of key epi-marks known to regulate stem cell differentiation^30^,^31^. In this report, we demonstrate the efficacy of the descriptors of the epi-marks to (i) clearly parse stem cells of varying lineage progression, and (ii) elucidate active differentiation events and predict stem cell lineage outcomes in defined microenvironments. Thus, the EDICTS methodology can be broadly applied to single-cell analysis of lineage decisions in developmental contexts for applications in regenerative medicine.

## Results

### Development of EDICTS: Optical High Content Nanoscopy of Bivalent Epi-Marks and Texture Informatics

**Figure 1:**
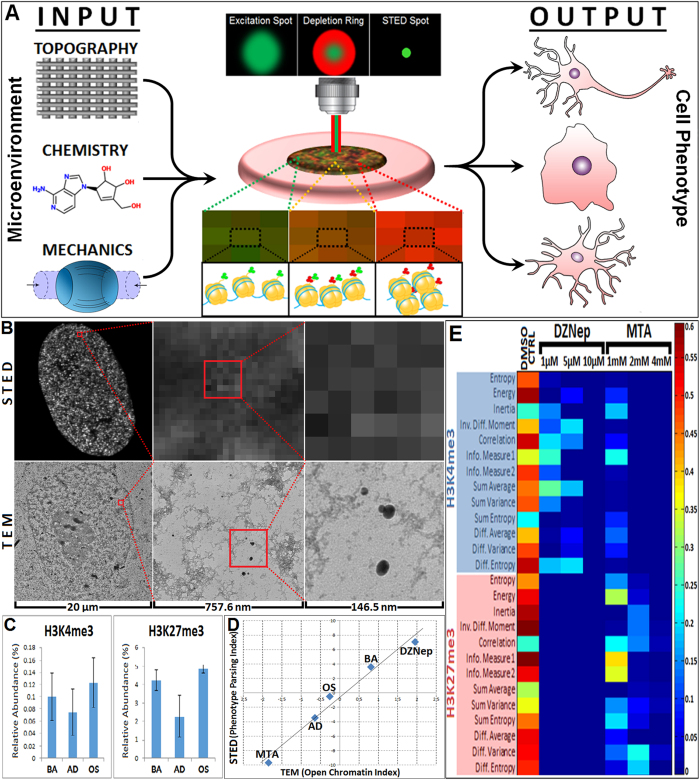
Schematic and Calibration of EDICTS (Epi-mark Descriptor Imaging of Cell Transitional States). (**A**) Workflow schematic of texture based high content image analysis of nuclear structure signatures. Undifferentiated stem cells are exposed to defined environmental cues and high resolution images of their immunolabeled nuclei are subsequently acquired via STED confocal nanoscopy. Haralick texture descriptors are extracted from these images via quantifying neighboring pixel relationships from grey-level co-occurrence matrices (GLCMs). The resolution of single pixel units correspond to the occupation of 1–4 nucleosomes. (**B**) Size scale relationship between high-content confocal images obtained with STED nanoscopy and electron micrographs obtained with TEM. Quantifications obtained from these images correspond to the dynamics of the gene transcription regulating bivalent mark H3K4K27me3. (**C**) Mass spectrometric quantification of H3K4me3 and H3K27me3 reveals their relative abundances are not significantly altered in response to soluble growth factors and differentiation. Lack of statistical significant differences determined by one-way ANOVA (K4me3: F(4,10) = 0.537235, p = 0.712008; K27me3: F(4,10) = 1.188573, p = 0.373457). (**D**) Correlation between LCSM and TEM quantitative datasets. A positive correlation between the “open chromatin index” from TEM micrographs and “phenotype parsing index” from LCSM images is apparent, with negative values of both indices corresponding to relatively closed chromatin states in post-mitotic cells and positive values of both indices corresponding to relatively open chromatin in undifferentiated stem cells. DZNep and MTA treated cells correspond to their positive and negative extremes, respectively. (**E**) Heat map of p-values from T-Tests computed between texture descriptor datasets of untreated and KMTi treated hMSCs reveals the sensitivity of all 104 texture descriptors to increasing KMTi concentration.

The overall workflow of the EDICTS encompasses four major steps related to nanoscopy of epi-marks: (i) antibody labeling of sentinel chromatin epi-marks in stem cells; (ii) super-resolution nanoscopy to image their nucleus-wide localization, (iii) image processing and bioinformatics analysis of textural features associated with the combinations and differences of two key epi-marks, and (iv) calibration of epi-mark texture descriptors with cell phenotypic variations (Fig. 1).

The key epi-marks selected in this report were the co-occuring trimethylations of lysine residues 4 and 27 on histone 3 (H3K4K27me3)^30^,^31^. These epi-marks are bivalent at the promoters of many key developmental regulators in undifferentiated stem cells and often resolve to monovalent states upon lineage commitment^31^. To calibrate the organization of these histone epi-marks at the nanometer scale, we used immunoelectron microscopy (IEM) to quantify the distribution of H3K4K27me3 in euchromatin versus heterochromatin nuclear regions. Secondary gold nano-bead antibodies were used to label single epi-marks in the nuclei of three different phenotypes of hMSCs and their density was quantified. Levels of active (H3K4me3) and silencing (H3K27me3) epi-marks were found to show distinct distributions in euchromatin and heterochromatin regions in undifferentiated versus differentiating hMSCs, where undifferentiated hMSCs exhibited the highest levels of the active H3K4me3 mark in euchromatin areas and the lowest levels of the repressive H3K27me3 mark in heterochromatic areas, and the opposite effect was observed in differentiated hMSCs (Fig. 1C, Supplementary Figure 3).

Mass spectrometric analysis established that the total H3K4K27me3 levels were relatively invariant in three very distinct stem cell phenotypes (Fig. 1D). To verify the regulatory role of these marks during MSC differentiation, we employed chromatin immunoprecipitation (ChIP), which confirmed that both H3K4me3 and H3K27me3 are dynamically associated with important developmental genes vital for the proper specification of different lineages (Supplementary Figure 4) Taken together, ChIP and MS analysis suggests that although the global levels of H3K4me3 and H3K27me3 are not significantly altered in response to hMSC development, their distribution along the genome changes markedly. These results underscore the importance of developing methods to capture the redistribution of the marks in the native spatial context of a single cell.

Next, we explored various image-processing approaches to detect phenotype-specific distinctions in H3K4K27me3 organization, supported by the underlying hypothesis that H3K4K27me3 spatial organization reflects unique chromatin structural patterns that drive lineage-specific gene regulatory processes. By screening a number of different image pattern characterization algorithms, including Gabor and Haar wavelet filters, we found that Haralick texture based algorithms captured the most variability of the phenotype-specific organizational signatures of these epi-marks (Supplementary Figure 5). These features are calculated from grey level co-occurrence matrices (GLCMs) that are a function of the angular relationship between neighboring pixels as well as the distance between them^29^ (Supplementary Table 1), and thus are most sensitive to intranuclear organizational patterns, rather than gross geometric shape features. While the scale of STED images is approximately 3 orders of magnitude less than TEM images (i.e. 400X vs. 400,000X) (Fig. 1C), the quantitative data extracted is sensitive enough to detect phenotype-specific epi-mark reorganization (Fig. 1E). Thus, distinct H3K4K27me3 organizational patterns in super-resolved images of phenotype-specific nuclei could be quantifiably identified based on the relative presence and distribution of these two opposing epi-marks.

### Calibration of EDICTS: Texture Descriptor Datasets Using KMT inhibitors to Distinguish “Open” versus “Closed” Chromatin Structural States

To optimize our ability to calibrate contrasting patterns of spatial organization of the histone epi-marks, indicating a shift in balance between H3K4me3 and H3K27me3, we generated quantitative training datasets using pharmacologic modulators of histone methylation states. Thus, increasingly “open” versus “closed” chromatin structural states were engineered via the inhibition of specific lysine methyltransferases (KMTs).

The nuclei of KMT inhibitor treated versus non-treated cells were subsequently imaged via G-STED nanoscopy and used to generate training datasets of descriptors reflective of relatively “open” versus “closed” chromatin structure states. This was accomplished by treating hMSCs with KMTi’s of opposing targets, 3-Deazaneplanocin A (DZNep) and Deoxy-methylthioadenosine (MTA), fixing and co-immunolabeling these cells for H3K4me3 and H3K27me3, and subsequently extracting quantitative descriptor sets from their high resolution images. A total of 104 quantitative texture descriptors were acquired for each single nucleus, and a minimum of 50 nuclei were imaged per condition.

To assess the sensitivity of responsiveness of the quantitative texture descriptors, statistical *t*-test analysis was performed on the descriptor sets of KMTi treated cells vs. a DMSO vehicle control, and these p-values were plotted onto heat maps (Fig. 1F). Even at the lowest concentrations of KMTi treatment, the majority of texture descriptors were shown to be quite different from the DMSO control, with only a handful of descriptors with a p-value over 0.15 (i.e. correlation and information of correlations 1&2). The relatively low sensitivity displayed by these correlation-based descriptors suggests that they are particularly sensitive to linear patterns in a ROI that may not be relevant for epi-mark organizational patterns. The higher sensitivity of descriptors relative to local detections of heterogeneity (i.e. entropy), or lack thereof (i.e. energy), indicates that these are sensitive to chromatin condensation patterns corresponding to euchromatic versus heterochromatic areas, which may govern changes in transcriptional regulation and subsequent stem cell differentiation. The highest degree of KMTi exposure yields the most different organizational patterns of H3K4K27me3 expression from the untreated control, with virtually every descriptor displaying a p-value of less than 0.05. Thus, descriptor sets of nuclei exposed to the highest KMTi concentrations (10 μm for DZNep, 4 mM for MTA) were used to train a support vector machine classifier to distinguish these two opposing structural signatures, which we subsequently applied to our test sets of non-treated differentiating cells.

These large descriptor datasets were dimensionally reduced using principal component analysis (PCA) to compute three principal components based on a linear transformation of all 104 descriptors. A “phenotype parsing index” value was computed from the coefficients of the eigenvectors used to linearly transform each of the three principal components, which enabled the average 3D spatial coordinates of cells exposed to specific stimuli to be referenced to a single number identifier along this index. This index was subsequently related to an “open chromatin index” computed from the quantification of the ratio of the active marks (H3K4me3) in euchromatin to silencing marks (H3K27me3) in heterochromatin from the IEM analysis. The correlation between these two indices supports the relation between the quantitative texture descriptor datasets extracted from nanoscale STED images and the nano-scale quantification via the TEM images (Fig. 1E).

### EDICTS Reveals Descriptors that Parse Early Stem Cell Differentiation Trends

**Figure 2:**
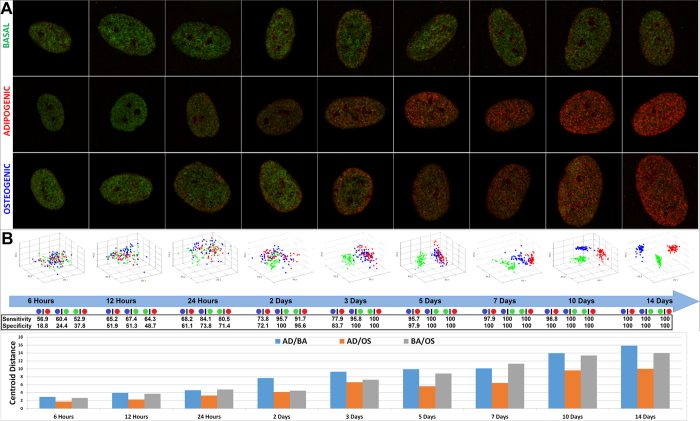
EDICTS captures the evolution of stem cell lineage restriction in time, as evidenced by four dimensional spatiotemporal image informatics of H3K4K27me3 expression in developing hMSCs. (**A**) Cells were either maintained in a basal stem state or induced to differentiate toward adipogenic and osteogenic lineages. hMSCs were subsequently fixed, immunolabeled and imaged for H3K4me3 (green) and H3K27me3 (red) at 9 time points over two weeks. All images shown are 30 μm × 30 μm. (**B**) Principal component analysis (PCA) of their texture descriptors reveals the ability to classify the three conditions as early as 72 hours post seeding, with classification statistical accuracy improving over each time point. An average sensitivity/specificity value above 80% indicates high classification accuracy, which is achieved at the 72 hour mark between all cell types, and as early as between 24 and 48 hours between undifferentiated hMSCs and hMSCs induced to differentiate. The distances between the centroids of each cell phenotype steadily increases over time, with greater separation between undifferentiated hMSCs and either differentiated cell type (i.e. adipogenic and osteogenic) compared to adipogenic versus osteogenic, suggesting that (i) these texture descriptors directly correlate to “degrees” of phenotypic commitment, and (ii) generally distinguish undifferentiated cells from differentiated cells earlier than distinguishing different differentiated cell types from each other.

Since epi-marks have been shown to regulate developmental gene transcription, we investigated the early dynamics of H3K4K27me3 prior to the onset of significant downstream cellular phenotypic changes. hMSCs were differentiated towards adipogenic and osteogenic lineages, as previously described^21^, fixed along 9 time points over a 2 week period, immunolabeled for H3K4K27me3, imaged and analyzed (Fig. 2). A temporal shift in balance towards a higher H3K27me3:H3K4me3 ratio as hMSCs are induced to differentiate towards adipogenic or osteogenic lineages was apparent, compared to the basal control (Fig. 2A). Haralick texture descriptors were acquired for each cell in three distinct differentiation media and subsequently classified by reducing these large descriptor datasets to 3 dimensions using PCA. After applying PCA analysis to descriptor sets acquired from each time point, these three cell types were effectively classified from each other as early as 72 hours following induction (Fig. 2B), 2–4 days prior to significantly distinguishing lineage markers (Supplementary Figure 5). Classification statistics also improved over each time point, and *k-*means cluster analysis revealed increasing 3-D spatial distance of different cell phenotype clusters over each time point. Thus, these temporal studies demonstrate the ability to capture distinct patterns of H3K4K27me3 spatial organization indicative of early differentiation, significantly in advance of changes in lineage-specific downstream biomarkers.

### EDICTS Descriptors Annotate Epigenetic Progression of a Broad Spectrum of Human Stem Cells

To demonstrate the robustness and versatility of Haralick texture features for detecting phenotype-specific epi-mark organizational patterns, the nuclear distribution and organization of the surrogate epi-mark H3K4K27me3 in two classes of developmental human stem cell systems were investigated. Cell cycle synchronization eliminated potential artifacts generated from differences in epi-marks due to cell cycle phase differences^32^. Induced human pluripotent stem cells (iPSCs) were generated by the retroviral transfection of human foreskin fibroblasts (HFF-1) with a vector containing the Yamanaka factors^33^. These iPSCs were subsequently differentiated to neural stem cells (NSCs) using soluble growth factors, as previously described^34^. Induced Neuronal cells (iNs) were generated from iPSCs via lentiviral transfection with a vector that has been shown to directly convert fibroblasts to functional neurons^35^. Cellular identity was confirmed via immunolabeling iPSCs with SSEA4, NSCs with Nestin, and iNs with TUJ1 (Supplementary Figure 6A–C). The successful differentiation of hMSCs towards osteogenic and adipogenic lineages was confirmed with Fast Blue and AdipoRed staining, respectively, as previously described^21^ (Supplementary Figure 6D). Consistent with expected changes in H3K4K27me3 bivalency during cellular differentiation, Förster resonance energy transfer (FRET) and ChIP data identified a system-specific loss in bivalent marks with differentiation and gain of H3K4me3 at differentiation-promoting genes (Supplementary Figures 4 and 7), thus validating these experimental systems for subsequent demonstration of the high-content chromatin nanoscopy technique.

**Figure 3:**
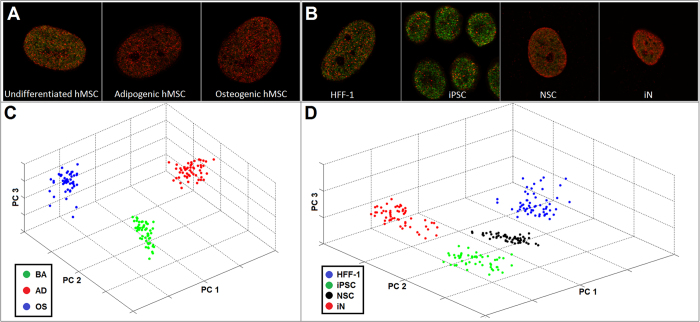
EDICTS can effectively parse distinct stem cell phenotypic states. H3K4K27me3 is dynamically regulated and differentially expressed throughout stem cell differentiation. (**A** and **B**) Nuclei of seven different distinct cellular phenotypes were immunocytochemically labeled for H3K4me3 (green) and H3K27me3 (red) and imaged with a LSCM G-STED. All images are 30 × 30 μm. (**C** and **D**) Quantitative texture descriptors of H3K4K27me3 organizational expression were extracted from these images and subsequently analyzed via PCA, which reveals the ability to clearly classify each different cellular phenotype from each other based on H3K4K27me3 textural expression with 100% sensitivity and specificity.

High content confocal images were acquired from cells that were immunocytochemically labeled using the same antibodies used in the ChIP assay (Fig. 3A and B). Based on the expression of H3K4K27me3, functionally distinct phenotypes were clearly classified from each other via Haralick texture descriptors with 100% statistical accuracy (i.e. 100% specificity and sensitivity), in both developmental cell systems between all seven phenotypes (Fig. 3C and D). In contrast, cellular phenotypes could not be parsed by Hoescht labeleing (Supplementary Figure 8A–D) nor nucleoli feature image analysis (Supplementary Figure 8E,F), indicating the unique ability of H3K4K27me3 organization as a surrogate marker for cellular development. Further, cells that were transfected with a lentivirus (i.e. iPSCs, NSCs and iNs) are clearly distinguished from HFF-1s, suggesting that the H3K4K27me3 expression of these cells annotates the phenotypic alteration of these cells (Fig. 3D).

### Role of Biophysical Microenvironment on Epi-mark Descriptors and Stem Cell Differentiation

Mechanical and topographical properties of substrates can sensitively modulate the differentiation of different cell types^36^,^37^,^38^, but the epigenetic constraints on early lineage restriction imposed by such substrates remain to be delineated. We applied the EDICTS approach to elucidate H3K4K27me3 signatures of cells cultured on materials with defined, graded mechanical and nanotopographical properties.

**Figure 4:**
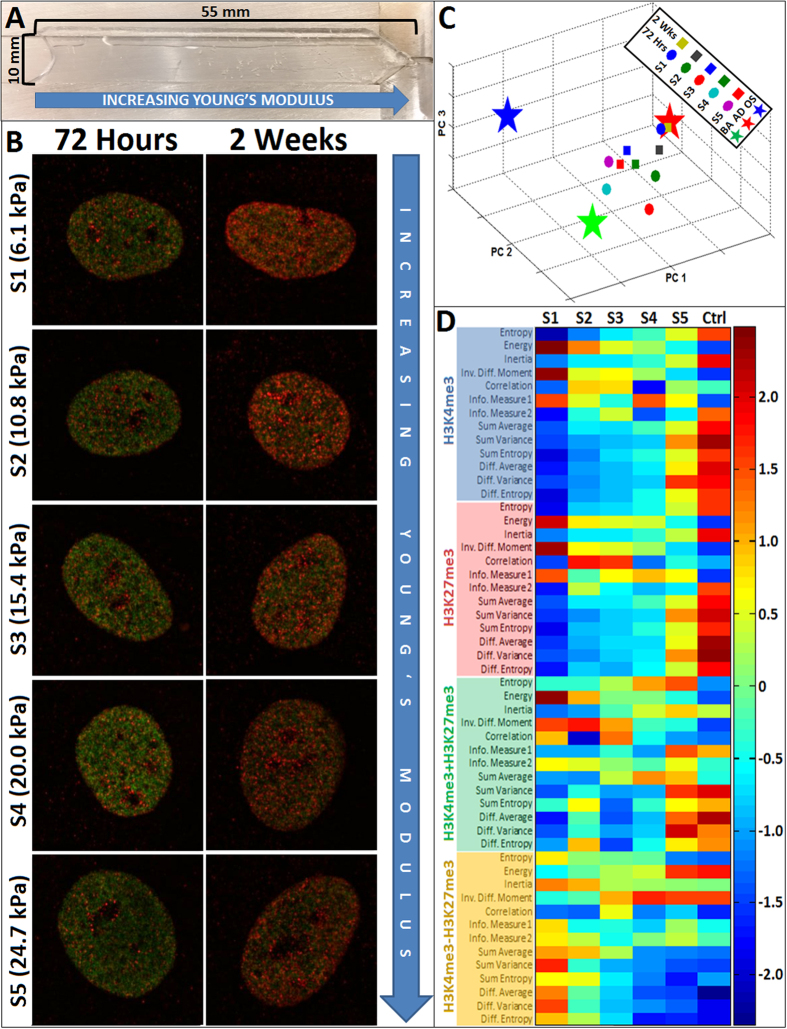
EDICTS resolves stem cell phenotypes steered by graded extracellular mechanics. H3K4K27me expression is responsive to minute changes in Young’s modulus. (**A**) PEGDM continuous Young’s modulus gradient hydrogels were fabricated by varying the dispensing rates of different PEGDM concentration solutions into a custom mold, yielding a final hydrogel “strip” measuring ~10 mm in width and ~55 mm in length. (**B**) hMSCs cultured on PEGDM continuous Young’s modulus gradient hydrogels were fixed and immunolabeled for H3K4me3 (green) and H3K27me3 (red) at early (72 hours) and late (2 weeks) time points along the entire length of the hydrogel, which ranged in Young’s modulus from 3.8 to 27 kPa. (**C**) Haralick texture descriptors were acquired and analyzed via PCA followed by k-means cluster analysis to determine centroids of clusters of cells cultured for a specified time and position on the gradient. Centroids of cells grown on the softest part of the gel (S1) were closest in proximity to the centroid of pre-differentiated adipogenic cells, indicating their similarity of H3K4K27me3 expression and subsequent lineage commitment. (**D**) Heat-map of normalized texture descriptor values reveals increasing (outlined in red) and decreasing (outlined in blue) trends that vary with decreasing hydrogel stiffness.

First, we investigated the role of substrate mechanical compliance on stem cell development using a polyethylene glycol dimethacrylate (PEGDM) hydrogel system exhibiting a spatially continuous gradient of stiffness from 3.8 to 27 kilopascals (kPa) (Fig. 4A and Supplementary Figure 10) containing a constant concentration of the cell adhesion peptide RGD^39^. hMSCs were cultured in a 50:50 cocktail of osteogenic:adipogenic induction media and fixed at early (72 hours) and late (2 weeks) time points, after which they were immunocytochemically labeled for H3K4K27me3 and prepared for high content STED image acquisition and analysis (Fig. 4B). The PEGDM substrates were grouped into five equal sized ranges of stiffness, and texture descriptor datasets were extracted from images of nuclei cultured on each of these regions. K-means cluster analysis was subsequently performed to determine the centroids of the descriptor sets of cells from each region, and these centroids were subsequently plotted to compare to the centroids of pre-differentiated hMSCs (Fig. 4C). Centroids of cells cultured on the softest region of the gel (S1) were closest in proximity to the centroid of pre-differentiated adipocytes, even at the early timepoint (72 hours), which also correlated with the highest levels of intracellular triglyceride staining (Supplementary Figure 5). Centroids of hMSCs cultured on increasingly stiffer regions of the gel were located further from the centroid of adipogenic hMSCs, with more pronounced differences at the early time point (Fig. 4B). Interestingly, no centroids seem to be in close proximity of the osteogenic hMSC centroid, suggesting the weak osteogenicity of this hydrogel system. All texture descriptor values were averaged for each hydrogel section and normalized, and subsequently plotted onto a heat-map to identify potential trends and compare with descriptors of pre-differentiated phenotypes. The majority of texture descriptors exhibit either an increasing or a decreasing trend that varies with decreasing PEGDM hydrogel stiffness (Fig. 4D). Further, the normalized descriptors of hMSCs cultured on the softest part of the gel (S1) approach values that are closest to those of pre-differentiated adipogenic hMSCs. Consistent with the histological lineage marker staining assays (Supplementary Figure 11), these results indicate that the textural profile of H3K4K27me3 expression during hMSC development can be used for screening and interpreting defined mechanical properties of biomaterials.

**Figure 5:**
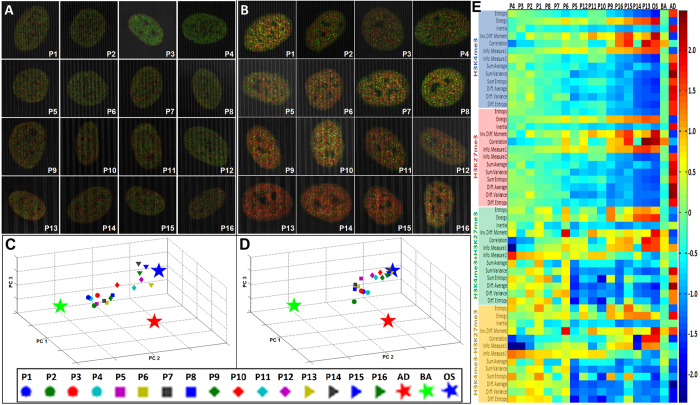
EDICTS can demarcate stem cell states on graded nanoscale substrate topographies. H3K4K27me3 expression is responsive to varied topographic line/space patterns. (**A** and **B**) LSCM G-STED images of hMSCs cultured on PUA topographic line/space patterns labeled for H3K4me3 (green) and H3K27me3 (red) reveal a shift in balance from H3K4me3 to H3K27me3 as line/space widths widen that can be qualitatively observed after 2 weeks (**B**), but less apparent earlier after 72 hours (**A**). (**C** and **D**) K-means cluster analysis reveals that centroids of hMSCs seeded on the widest line/space patterns (triangles) are closest in proximity to osteogenically differentiated hMSCs after 72 hours (**C**) and migrate closer after 2 weeks (**D**), whereas centroids of hMSCs cultured on the narrowest line/space patterns (circles) are closest in proximity to undifferentiated hMSCs after 72 hours (**C**), but gradually migrate closer towards osteogenic after 2 weeks (**D**). (**E**) Heat-map of normalized texture descriptor values reveal increasing and decreasing trends that vary with increasing line/space width (left to right). The correlation and information of correlation 1 descriptors increase with line/space width, whereas the information of correlation 2 descriptor decreases, which is a trend not previously seen on hMSCs cultured on PEGDM hydrogels, indicating a subset of “material-specific” descriptors.

Next, the role of substrate nanotopography on chromatin structural dynamics was characterized via EDICTS. A substrate consisting of 16 different defined topographical patterns was fabricated via the soft lithography of polyurethane acrylate (PUA). These patterns consist of systematically varied line/space widths, ranging from 350 nm to 2000 nm (Supplementary Figure 12). hMSCs were seeded onto these “nano-grooves” and cultured in a 50:50 cocktail of osteogenic and adipogenic induction media, after which they were fixed at early (72 hours) and late (2 weeks) time points, and subsequently immunolabeled for G-STED imaging and high content image analysis (Fig. 5A and B). K-means cluster analysis was performed to determine the centroids of cells cultured on each of the 16 patterns, and these centroids were plotted in relation to those of pre-differentiated hMSCs (Fig. 5C and D). The K-means plots indicate that centroids from cells cultured on increasingly wider line/space widths were located proximal to the centroid of pre-differentiated osteogenic hMSCs, even at the early time points (Fig. 5C), and this trend was even more pronounced at the later time point (Fig. 5D). Heat-maps of normalized descriptor values on each topographical pattern reveal correlating trends with widening line/space patterns, with values approaching those of pre-differentiated osteogenic hMSCs on the widest patterns. (Fig. 5D). Moreover, the correlation texture descriptors vary consistently with nano-scale changes in line/width spacing, which was not previously seen in cells cultured on the PEGDM hydrogel, nor in any cells analyzed in 2D. This may indicate a manifestation of H3K4K27me3 that is sensitive to linear patterns when cultured on this substrate, consistent with the linear topographic patterning of this substrate. These findings correlate with the late time point bone-specific alkaline phosphatase staining patterns obtained in parallel (Supplementary Figure 13). Further, no descriptor centroids of hMSCs grown on these nano-grooves were in close proximity with those of adipogenically pre-differentiated hMSCs, indicating that their H3K4K27me3 signatures are quite different. This also corroborates with the lack of intracellular tri-glyceride staining and fat-globule formation from hMSCs cultured on this substrate (Supplementary Figure 13), as well as a lack of similarity with the normalized descriptor values (Fig. 5D). Thus, the EDICTS method for organizational mapping of H3K4K27me3 can sensitively screen mechanical and topographical properties of biocompatible substrates for insights on cellular development for applications in regenerative medicine.

## Discussion

While investigations of the “histone code” have advanced our understanding of genome wide epigenetic regulation of transcriptional activity^11^,^40^, single cell level elucidation of epigenetic activity remains a challenge. By integrating optical super-resolution nanoscopy and high dimensional texture analysis of chromatin epigenetic modifications, EDICTS offers a new image informatics methodology to annotate single cell epi-mark territorial organization. We report that EDICTS offers a way to fingerprint single-cell epimark organization and establishes the first such reported link between the organization of bivalent epi-marks and degree of stem cell lineage differentiation.

Previously, we reported on the ability to demarcate differentiating mesenchymal stem cells based on geometrical features of cytoskeletal organization, in response to a variety of chemical factors^21^. However, this approach is not able to accurately probe the effect of biophysical factors on differentiation, since the overwhelming effect of physical factors on cytoskeletal organization largely trumps any potential insights on lineage commitment. Since geometrical aspects of nuclei are largely unaffected by biophysical manipulation, we investigated ways to exploit unique nuclear responses to external stimuli to provide clues into cellular behavior.

The efficacy of EDICTS relies on the pixel-by-pixel quantification of spatial dependence matrices of the organizational patterns of the fluorescently labeled epi-marks (e.g., bivalent epi-marks, H3K4me3 and H3K27me3). EDICTS yielded sensitive information about organizational patterns of epi-marks that were expressed at low levels as quantified by population-level proteomic mass spectrometry (Fig. 1D). Even these low frequency epimarks can reorganize to a striking degree in early stages of stem cell lineage restriction, correlating ultimately with distinct gene transcription programs and phenotypes. While this study was limited to an investigation of H3K4K27me3, EDICTS has much broader relevance to a host of other nuclear markers whose textural organization could potentially provide insight into the behavioral state of a cell, especially markers intimately linked to the formation of heterochromatin and gene silencing, such as heterochromatin protein 1-alpha (HP1α)^41^,^42^. It should be noted that a parallel high content analysis of conventional nuclear morphology via stains DAPI and Hoechst 33342, failed to accurately characterize and classify cells (Supplementary Figure 8). Further, macro-scale descriptors relevant to gross geometric features acquired from H3K4K27me3 labeled nuclei^21^ also lacked the ability to accurately classify different cell phenotypes. This suggests that geometric nuclear descriptors could not robustly classify cells in the absence of features that embody intranuclear organizational patterns.

Because EDICTS relies on resolving intranuclear organizational textures of epi-marks, the optical resolution of the images is critical to the efficacy of EDICTS. Here, we demonstrate that G-STED microscopy (resolution < 50 nm) was clearly more effective than conventional confocal microscopy to discern variations in epi-mark organization (Supplementary Figure 1). The resolution of conventional confocal microscopy is limited by Abbe’s diffraction barrier, which is generally on the order of 200–300 nanometers. By employing a stimulated emission depletion laser, we are able to improve this resolution to about <50 nm, which enables the resolution of 1–5 individual nucleosomes per pixel. To establish the inverse effect of “coarsening” the resolution on cell phenotype parsing, when a median filter was applied to progressively lower the digital resolution, different cell phenotypes were increasingly harder to distinguish with increased “blurring” based on this intranuclear organization (Supplementary Figure 2).

One of the key applications of EDICTS is the ability to directly compare the organizational signatures of H3K4K27me3 of cells cultured on different engineered materials against those that were pre-differentiated. The addition and loss of epigenetic marks are often upstream of the gene transcription programs necessary for proper phenotypic development. Therefore, profiling H3K4K27me3 dynamics in response to external stimuli can give insight into the early behavioral state of the cell. Quantitatively analyzing these changes using descriptors mapped in a common analytical space (i.e. PCA plot) enables the ability to directly compare known epigenetic profiles with those that are responding to various, defined external stimuli. Specifically, the k-means cluster analysis of hMSCs grown on materials with graded mechanical compliance or topographies yielded very different H3K4K27me3 organizational patterns that were predictive of their ultimate lineage commitment. These trends were evident at early time points (within 72 hours of culture), prior to the manifestation of distinguishable downstream markers such as intracellular triglyceride and alkaline phosphatase expression for fat and bone lineages, respectively (Figs 4 and 5 and Supplementary Figures 11 and 13). Thus, EDICTS can be applied to forecast lineage states and screen microenvironmental properties that may steer stem cell lineage commitments. Additionally, we observe that different epigenetic constraints (e.g., nanotopography, mechanical compliance, growth factor stimulation) produce specific texture descriptor groups as the more dominant traits (see Supplementary Table 3). These dominant traits can be identified from the principal component analysis as the highest eigenvector coefficients that make up the key principal components. For example, epimark “uniformity” family of texture descriptors emerge influential under high growth factor stimulation conditions, whereas “correlational metrics” are more influential under more dominant nanotopographic constraints and weakly influential for mechanically stiff substrates and growth factor conditions. The physical attributes of these descriptors are discussed further in Supplementary Figure 9. Because topographic constraints affect the nuclear mechanoskeletal biology more anisotropically, we expect that epimark organizational heterochromatic regions tend to aggregate along the perimeter of a nucleus along the nuclear membrane^43^,^44^, thus yielding local correlations of epi-mark textures forming along the nuclear perimeter as a stem cell differentiates.

The datasets from EDICTS can be used to potentially complement other higher resolution image techniques and *in silico* modeling simulations on chromatin structural dynamics, for example, studies of specific factors that are directly involved in nucleosome assembly^45^, chromatin remodeling^46^,^47^, or target chemically modified histone tails^48^,^49^ or methylate DNA^50^, and subsequently link the resultant structural dynamics to altered gene transcription patterns that ultimately drive different cellular behaviors^51^. Biochemical investigations of gene interactions via chromatin conformation capture^52^,^53^ and studies of spatial organization of chromosomes via 3D fluorescence *in situ* hybridization (FISH) also provide complementary, cell population-level insights^54^,^55^,^56^. To our knowledge, this study is the first to report on the existence and detection of unique intranuclear histone PTM organizational patterns reflective of dynamic chromatin structures that correlate with specific phenotypic commitment in intact stem cells.

Beyond the power of EDICTS to forecast lineage development, the epi-mark descriptor datasets can also provide new biological insights that could be relevant to epigenetic signaling mechanisms. In the future, the epi-mark dynamics could be studied in concert with the localization dynamics of other chromatin remodeling protein groups, such as polycomb^57^, trithorax^58^ and SWI/SNF complexes^59^. We also observe that the epi-mark texture information is particularly sensitive for cellular parsing when aggregated globally across a cell (Supplementary Figure 14). It would be interesting to probe whether local domains of pronounced textures exist within a cell, or whether this information is globally coordinated, and what molecular mechanisms and communications exert control over these phenomena. Further, the development of viral based fluorescent probes targeting specific epi-marks can enable live-cell monitoring of their organizational dynamics in real time, which can be used to potentially sort cells after classification.

In summary, Epi-Mark Descriptor Imaging of Cell Transitional States (**EDICTS**) is a high-content textural image analytical tool that is able to detect surrogate signatures of chromatin structural dynamics and their influence on emergent cell phenotypes. As the markers we investigated are epigenetic and are globally present in every human cell type, this technique can be a powerful tool in the early assessment of stem cell development for the improved efficiency and efficacy of generating any lineage restricted phenotype of interest.

## Methods

### hMSC Sourcing, Culture and Directed Differentiation

Purified hMSCs were obtained frozen from the Tulane University Center for Gene Therapy (Donor: 7071L), thawed and cultured in α-MEM containing 10% FBS and 0.5% Penicillin/Streptomycin (Invitrogen) in a water-jacketed incubator kept at 37 °C and 5% CO_2_. Basal culture media was changed every 72 hours until cells reached 70% confluency, at which point they were passaged into fresh flasks or dishes at a seeding density of 5,000 cell/cm^2^.

Adipogenic hMSCs were generated by supplementing the basal culture media with soluble growth factors that promote adipogenic differentiation. Two media formulations were involved: Adipogenic induction media (AIM) and adipogenic maintenance media (AMM). AIM consisted of basal culture media supplemented with 1 μM Dexamethasone (Sigma-Aldrich), 50 μM Indomethacin (Sigma-Aldrich), 10 μg/ml Insulin (Sigma-Aldrich) and 100 μM 3-Isobutyl-1-methyl-xanthine (Sigma-Aldrich). AMM consisted of basal culture media supplemented with 10 μg/ml Insulin. Adipogenic hMSCs were generated over a two week culture period, with differentiation beginning when hMSCs reached ~100% confluency, at which point the basal culture media was replaced with AIM and incubated for 72 hours. Next, AIM was replaced with AMM and incubated for 48 hours. AMM was then replaced with AIM for another 72 hours, then replaced with AMM for another 48 hours. AIM and AMM was alternated as such for two weeks.

Osteogenic hMSCs were generated by supplementing the basal culture media with 0.5 mM L-Ascorbic Acid-2-Phosphate (Sigma-Aldrich), 20 mM β-glycerol phosphate (Sigma-Aldrich) and 0.2 μM Dexamethasone. Differentiation induction began 24 hours after plating undifferentiated hMSCs at a seeding density of 3,000 cell/cm^2^, by replacing the basal culture media with osteogenic media, which was subsequently replaced every 72 hours over a two week period.

### HFF-1 Sourcing, Reprogramming and Directed Differentiation

Human foreskin fibroblasts (HFF-1) were obtained from the Rutgers University Cell and DNA Repository (RUCDR) and cultured in DMEM (Life Technologies) supplemented with 2 mM L-glutamine, 10% FBS, 1% Non-essential amino acids (Life Technologies) and 1% Penicillin/Streptomycin. Induced pluripotent stem cells (iPSCs) generated from HFF-1s by retroviral transfection with OCT4, SOX2, Klf4 and c-Myc, as previously reported were also a gift of the RUCDR^33^. After selection and purification, iPSCs were cultured on Matrigel (BD Biosciences, San Jose, CA, USA) treated culture dishes in the defined medium mTeSR-1 (Stem Cell Technologies, Vancouver, CA), and this media was changed every 24 hours.

Neural stem cells (NSCs) were generated from iPSCs by replacing their mTeSR culture media with an N2 conversion medium which consists of 50% DMEM/F12 (Life Technologies), 50% Neurobasal Media (Life Technologies), 2 mM L-glutamine, 0.5X N2 Supplement (Life Technologies), 0.5X B27 Supplement w/o Vitamin A (Life Technologies), 1% Penicillin/Streptomycin and 20 ng/ml basic fibroblast growth factor (Sigma-Aldrich) and this media was replaced every 24 hours. After 2 weeks of neuronal induction, media was changed to neural differentiation media, which consists of Neurobasal media supplemented with 1X B27 Supplement w/o Vitamin A, 1% Penicillin/Streptomycin, and 10 ng/ml brain derived neurotrophic factor (PeproTech, Rocky Hill, NJ, USA).

Induced neuronal cells (iNs) were generated from both iPSCs and HFF-1s via retroviral transfection, as described previously, using protocols approved by Rutgers Institutional Biosafety committee^35^. Briefly, iPSCs or HFF-1s were transfected in a growth medium containing 8 ug/ml polybrene with rtTA plus Brn2, Ascl1, Myt1l and NeuroD1. After 24 hours of transfection, media was replaced with growth media supplemented with 2 ug/ml doxycycline (Sigma-Aldrich). After an additional 24 hours, media was replaced with N2 conversion media for a period of 6 days, and then replaced with neural differentiation media. *The generation and sourcing of human stem cells were covered by respective IRB institutional protocols at other centers (RUCDR; Tulane Center for Gene Therapy). The specific handling of adult human stem cells was carried out in accordance with relevant guidelines and regulations of the Rutgers Environmental and Health Safety (REHS); and the experimental protocol (12–155) approved by Rutgers Institutional Biosafety Committee (IBC) specifically covered the lentiviral generation of human induced neuronal cells.*

### Cell Cycle Synchronization

Cell cycle synchronization of all cell types was achieved using an early S-phase block by applying Thymidine (Sigma-Aldrich) in two cycles to allow cells to progress synchronously through the G2 and mitotic phase. Briefly, at about 30% confluency, each respective culture media was supplemented with 2 mM Thymidine and applied for 18 hours. Then the Thymidine was removed and replaced with normal culture media for 9 hours to release the cells from the S-phase block, after which 2 mM Thymidine was reintroduced to the cells for 17 hours. Cells were subsequently washed in PBS and incubated back in their normal culture media, where they were allowed to grow until ready for analysis.

### Osteogenic and Adipogenic Cellular Differentiation Assays

The generation of adipogenic and osteogenic cells was assessed using staining assays for intracellular triglyceride droplet formation and bone-specific alkaline phosphatase activity, respectively. Briefly, intracellular triglyceride droplets were stained with AdipoRed assay reagent (Lonza, Allendale, NJ, USA) by following the manufacturer’s directions. Positively stained adipogenic hMSCs were imaged using a Nikon Eclipse TE2000-S fluorescence microscope (Nikon Instruments, Melville, NY, USA) under a G-2A longpass emission filter covering 510–560 nm. Bone-specific alkaline phosphatase activity was detected using the fast blue rapid release alkaline phosphatase kit (Sigma-Aldrich) and following the manufacturer’s directions. Positively stained osteogenic hMSCs were imaged with the same microscope under bright field. Cell were also labeled with Hoechst 33342 (Life Technologies) via incubation in a 0.01 mg/ml solution for 10 minutes. All quantifications were made by measuring the Density/Intensity Sum values using Image Pro Plus 7.0 (Media Cybernetics, Rockville, MD, USA), and then normalizing to cell number by dividing by Hoechst positive nuclei.

### Lysine methyltransferase inhibition

hMSCs and HFF-1s were treated with lysine methyltransferase inhibitors (KMTi) to forcibly assume a chromatin structural state that is either more open or more closed. Cells were treated with 1, 5 and 10 μM of 3-Deazaneplanocin A (DZNep) (Cayman Chemical, Ann Arbor, Michigan, USA) for 72 hours to selectively inhibit the enzymatic activity of EZH2, which is a KMT that targets lysine residue 27 on histone 3. 1, 2 and 4 mM of 5′-Deoxy-5′-(methylthio)adenosine (MTA) (Sigma-Aldrich) was introduced to cells for 24 hours to inhibit the MLL family of enzymes, which catalyze the trimethylation of lysine residue 4 on histone 3. After drug treatment, cells were subsequently fixed in 4% Paraformaldehyde (Electron Microscopy Sciences, Hatfield, PA, USA) and prepared for immunocytochemical labeling.

### Western Blot

The inhibitory efficacy of the KMTi’s was determined via immunoblot analysis. Following KMTi treatment, cells were lysed on ice using RIPA lysis buffer (Thermo Scientific, Waltham, MA, USA) and harvested via scraping. Raw cell lysates were clarified via centrifugation at 15000 RPM, 4 °C for 15 minutes. Supernatant was subsequently collected and flash frozen in liquid nitrogen until ready for analysis. Protein quantification was determined using the BCA protein assay (Thermo Scientific) by following the manufacturer’s instructions.

After quantification, samples were heated on a heating block preheated to 95 °C for 10 minutes. Samples were subsequently diluted in the appropriate amount of RIPA buffer to load the same amount of protein (15 μg) into each well, and 5 μl of loading dye was added to each sample. An electrophoresis unit (Bio-Rad, Hercules, CA, USA) was assembled with a 7.5% polyacrylamide gel (Bio-Rad) immersed in 1X SDS Running Buffer consisting of 25 mM Tris (Bio-Rad), 192 mM glycine (Fisher Scientific, Pittsburgh, PA, USA) and 0.1% Sodium dodecyl sulfate (Sigma-Aldrich). After each well was loaded with protein and dye, the electrophoresis unit was allowed to run for 1 hour at 140 V. Next, a gel holder cassette was assembled with two sponges, four filter papers (Whatman plc, Maidstone, Kent, UK), nitrocellulose paper (Bio-Rad) and the gel. This cassette was placed into a transfer tank (Bio-Rad) and filled with 1X Transfer Buffer consisting of Tris/Glycine Buffer (Bio-Rad) supplemented with 20% Methanol (Sigma-Aldrich) and allowed to run for 2 hours at 300 mA at 4 °C while stirring. The membrane blot was subsequently removed and placed in Blocking Buffer consisting of 5% nonfat milk in 1X TBS-T for 1 hour at room temperature with gentle rocking. Membranes were then cut according to a reference protein ladder (Fisher Scientific) and then incubated in the primary antibodies H3K4me3 (Abcam, Cambridge, MA, USA), H3K27me3 (Abcam) and the control GAPDH (Santa Cruz Biotechnology, Dallas, TX, USA) diluted in a blocking solution (5% Bovine Serum Albumin in TBS-T) overnight at 4 °C on a rocker. The following day, membranes were washed with TBS-T for 10 minutes, four times. Then the membranes were incubated in HRP-conjugated secondary antibodies (Santa Cruz Biotechnology) diluted in a blocking solution (5% BSA in TBS-T) for 2 hours at room temperature, on a rocker. The membrane was then washed with TBS-T for 10 minutes, four times. After washing, the HRP detection solution, Luminata Forte Western HRP Substrate (Millipore, Billerica, MA, USA), was added for 5 minutes. Finally, the membranes were developed with autoradiography film (Denville Scientific, South Plainfield, NJ, USA) in a dark room using a Kodak X-Omat 2000A Processor (Kodak, Rochester, NY, USA).

### Immunoelectron Microscopy

Nuclei from cultured cells were isolated using the Nuclei EZ Prep Kit (Sigma-Aldrich) by following the manufacturer’s instructions. Nuclear pellets were fixed in 4% paraformaldehyde (Electron Microscopy Sciences) and 0.25% glutaraldehyde (Electron Microscopy Sciences) in PBS for 1 hour at room temperature. Fixative was subsequently removed and rinsed in dH_2_O before dehydration in a concentration series of ethanol (50%, 70%, 85%, 95%, 100%, for 10 minutes each). 100% ethanol was removed and replaced with a solution consisting of 50% ethanol and 50% LR White resin (Electron Microscopy Sciences) for 1 hour. This solution was subsequently removed and the pellet was incubated in 1 ml of 100% LR White resin at 60 °C overnight. After polymerization, 75 nm sections were cut using a Leica EM UC6 ultramicrotome and subsequently transferred onto formvar-carbon copper grids. Residual aldehydes were deactivated via incubation in 0.1 M glycine for 15 minutes. Sections were then permeabilized in 0.1% Triton-X100 in PBS for 5 minutes, and then blocked with 5% normal donkey serum (Abcam) for 1 hour at room temperature. After blocking, sections were incubated in primary antibodies for H3K4me3 (cat. no. ab8580, Abcam) and H3K27me3 (cat. no. ab6002, Abcam) at a 1:20 dilution ratio for 2 hours at room temperature. Sections were then washed in 5% normal donkey serum three times for 5 minutes each wash. Sections were subsequently incubated in secondary Aurion immunogold antibodies sized at 6 and 10 nm (Electron Microscopy Sciences) at a 1:30 dilution ratio for 2 hours at room temperature. Sections were then washed in PBS four times for 5 minutes each wash, and then post-fixed in 2.5% glutaraldehyde for 20 minutes. Sections were then washed in dH_2_O three times for 5 minutes each wash, and then stained with 2% uranyl acetate for 30 minutes followed by lead citrate for 30 seconds. Immunolabeled sections were subsequently imaged using a Zeiss LEO 912 transmission electron microscope.

### Immunocytochemical labeling

Cells were fixed in 4% Paraformaldehyde (Electron Microscopy Sciences) for 20 minutes at room temperature, then washed for 5 minutes with PBS w/o Ca2+ or Mg2+ (Lonza) three times. Next, they were permeabilized in a solution of 0.1% Triton X-100 (Sigma-Aldrich) in PBS for 10 minutes under gentle agitation, after which they were blocked with 8% Normal Goat Serum (Cell Signaling Technology, Danvers, MA, USA) for 1 hour. The primary antibodies H3K4me3 (cat. no. ab8580, Abcam) and H3K27me3 (cat. no. ab6002, Abcam) were diluted in 8% Normal Goat Serum solution at a 1:500 ratio and incubated at room temperature for 1 hour under gentle agitation. After incubation, cells were washed in PBS for 30 minutes at room temperature, three times. Secondary antibodies were diluted in 8% normal serum solution at a 1:1000 ratio and incubated at room temperature under gentle agitation for 2 hours, while protected from light. Secondary antibody solution was subsequently washed in PBS for 45 minutes, four times. After 3 total hours of washing, coverslips and PUA patterned substrates were mounted onto glass slides with ProLong Gold antifade mounting media (Life Technologies) and sealed along the perimeter with nail polish. Cells labeled on PEGDM hydrogels were not mounted, but stored in PBS and imaged by inverting onto MatTek glass bottom dishes (MatTek Corp., Ashland, MA, USA).

### Fӧrster Resonance Energy Transfer Analysis

FRET experiments were performed using the FRET Sensitized Emission Wizard bundled with the Leica Confocal Software version 2.61 build 1537 (Leica Microsystems, Buffalo Grove, IL, USA). First, the experimental conditions were set by defining laser power, photomultiplier tube detection range, and electronic gain and offset values for the donor and acceptor fluorophores. Next, calibration images were acquired from samples labeled with only the donor fluorophore and only the acceptor fluorophore, using the defined parameters that were set. Regions of interest (ROI) were defined by outlining areas of the image where ~100% of the ROI was occupied with donor signal, and background subtraction was performed. Finally, FRET samples were imaged and FRET efficiencies were computed based on the following equation (E1):


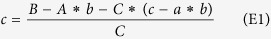


where A = Donor intensity, B = FRET intensity, C = Acceptor intensity, and a, b and c are the calibration factors determined from the donor alone and acceptor alone reference images, which are defined as: b = B/A, c = B/C, and a = A/C.

### High-content imaging

Immunocytochemically labeled samples were imaged using a Leica TCS SP8 confocal laser scanning microscope equipped with a time-gated stimulated emission depletion laser (G-STED) (Leica Microsystems). AlexaFluor 488 was visualized using a supercontinuum white light laser source set to 492 nanometer wavelength excitation and emission was detected using a hybrid detector (HyD) set to a range of 505 to 550 nanometers with a gain of 100 and offset of −6.667E-03, and time gating was set from 0.5 ns to 6.0 ns with a 499 nm gate reference wavelength. AlexaFluor 594 was visualized with 592 nanometer wavelength excitation, and emission was detected with a HyD set to a range of 600 to 655 nanometers with a gain of 267 and offset of −4.225E-03, and time gating was set from 0.3 ns to 6.0 ns with a 499 nm gate reference wavelength. The objective used for all high content image acquisition was a 100x Oil Immersion Objective with a numerical aperture of 1.44. Three dimensional z-stacks were taken with a pinhole of 0.75 airy units at a zoom of 5X to yield an XY physical dimension of 23.25 × 23.25 μm at a resolution of 1024 × 1024 pixels. Z-depth varied between 5–15 μm for different nuclei, and the optical section thickness remained constant at 0.5 μm.

### Computational methods of classification

Images were subsequently processed for analysis using background subtraction and object segmentation, to clearly isolate nuclear signals from any potential noise, using Image Pro 7.0 (Media Cybernetics, Rockville, MD, USA). Z-stack images were processed for average projection using Leica Application Suite Advanced Fluorescence software, and 2D average projections of z-stacks were used for Haralick texture image analysis. 42 Morphological descriptors included in the Image Pro software package were obtained for comparative purposes. 104 Haralick texture descriptors were acquired using a custom MATLAB script (Mathworks, Natick, MA, USA) written to compute different features based on nearest-neighbor spatial-dependence matrices. Thirteen total mathematically defined texture features (Supplementary Table 1) were computed for four different statistical values (mean, standard deviation, kurtosis, skew). These were based on spatial-dependence matrices of two varieties: the first combination was based on measurements made from spatial distances of one pixel, and the second combination was based on spatial distances of two pixels. Thus, a total of 13 × 4 × 2 = 104 different quantitative textural values were computed for each nucleus.

Haralick texture descriptors of KMTi treated cells were obtained and compared with those of untreated cells via Student’s T-TEST using Microsoft Excel 2010, and p-values were subsequently plotted onto a Heat Map using MATLAB (Mathworks). Descriptor sets of cells treated with the highest concentrations of each respective drug (DZNep - 10 μM, MTA 4 mM) were used to train a support vector machine classifier, which was subsequently used in all experimental PCA analyses. All PCA analyses were conducted using the Multivariate Data Analysis package, which is part of MATLAB’s Statistics Toolbox (Mathworks). Subsequent K-means cluster analysis was performed using the Cluster Analysis package, which is part of the same toolbox. The phenotype parsing index was computed from texture descriptor datasets extracted from confocal images by averaging the eigenvector coefficients along the three values of the XYZ coordinates of centroids determined from k-means cluster analysis. The open chromatin index was determined from IEM micrographs by computing the ratio of H3K4me3 positive marks to H3K27me3 positive marks in euchromatic regions, divided by the ratio of H3K27me3 positive marks to H3K4me3 positive marks in heterochromatic regions.

### Chromatin immunoprecipitation

To prepare for a single ChIP experiment, cells were expanded in culture to a minimum total of 1 × 10^6^ to yield a sufficient amount of purified chromatin. After expansion, cells were fixed in 1% formaldehyde in DMEM (Life Technologies) for 10 minutes at 37 °C, with gentle agitation every 2–3 minutes. Plates were then quickly washed in 1X PBS, two times, and cells were harvested via scraping in 1X PBS containing 1X Protease inhibitor (G-Biosciences, St. Louis, MO, USA) and collected in DNA Lobind tubes (Eppendorf, Hauppauge, NY, USA). Tubes were centrifuged at 600xG at 4 °C for 5 minutes, then supernatant was removed and cell material was resuspended in 300 μl of lysis buffer (10 mM EDTA, 50 mM Tris, 1X Protease Inhibitor and 1% SDS in DI H_2_O). At this point, samples could be flash frozen in liquid nitrogen and stored at −80 °C for future use. Sonication conditions were empirically determined for each cell type to yield chromatin fragments of 200–500 base pairs, and DNA fragment size was determined via electrophoresis on a 1% agarose gel run at 85 V for 1 hour. Reverse crosslinking of DNA and protein was achieved via heating at 65 °C for 6 hours.

Magnetic Protein G Dynal beads (Life Technologies) were transferred to DNA Lobind tubes which were placed on a magnetic rack (New England BioLabs, Ipswich, MA, USA) to separate beads from their solution. This solution was removed and replaced with 500 μl of 5% BSA in PBS and placed on a rotator for 15 minutes at 4 °C. Antibodies targeting proteins of interest were diluted in 5% BSA and incubated on a rotator for a minimum of 4 hours, or overnight, at 4 °C. After antibodies bound, the beads were washed in 5% BSA for 30 minutes, followed by a quick rinse.

Immunoprecipitation was achieved by diluting chromatin samples in dilution buffer (1X Protease Inhibitor, 20 mM Tris, 150 mM NaCl, 2 mM EDTA, 1% Triton in DI H_2_O) at a 1:7 ratio. Diluted chromatin was added to beads with bound antibody and allowed to incubate on a rotator at 4 °C overnight. The following day, DNA bound beads were washed in RIPA buffer for 5 minutes, five times, followed by a quick rinse in TE buffer. TE was removed and beads were suspended in a buffer consisting of 1% SDS and 10% NaHCO_3_. DNA was subsequently eluted off the beads and reverse-crosslinked from protein via heating at 65 °C for a minimum of 6 hours. After reverse-crosslinking, DNA was purified using the QIA quick PCR purification kit (Qiagen, Hilden, Germany, EU), by following the manufacturer’s instructions. DNA yield was quantified using the Quant-iT PicoGreen Assay (Life Technologies) by following the manufacturer’s instructions.

### Mass Spectrometric Quantification of PTMs

Histones were acid extracted as previously described^60^. Total histones were subjected to chemical derivatization using propionic anhydride and digested with trypsin for 6 hours at 37 °C at 10:1 substrate to enzyme ratio. The digested peptides were treated with an additional round of propionylation and desalted using C18 extracted mini disk (Empore 3 M, MN, USA). Approximately 1 ug of each sample was loaded via an autosampler (EASY-nLC. Thermo Fisher Scientific Inc) onto a homemade 75 μm reversed phase analytical column packed with C18-AQ resin (3 μm particle sizes, 120 Å pore size). Peptides were chromatographically resolved using a 66-min 2–98% solvent B gradient (solvent A = 0.1% formic acid, solvent B = 100% acetonitrile) at a flow rate of 300 nL/min. The electrosprayed peptides were detected by Orbitrap Elite mass spectrometer (Thermo Fisher Scientific Inc) with a resolution of 60,000 for full MS spectrum followed by MS/MS spectra obtained in the ion trap. The relative abundance of each modification, expressed as a percentage on a histone peptide sequence, was quantified by analyzing its MS and MS/MS spectra via an in-house software, EpiProfile.

### Quantitative Real Time Polymerase Chain Reaction

DNA oligonucleotide sequences (Supplementary Table 2) were designed to target slightly upstream of the promoter region of the genes of interest. These sequences were obtained from Integrated DNA Technologies (Coralville, IA, USA) as 25 nmole lyophilized powders, and resuspended in 250 μl of dH_2_O to yield 100 μM stock concentrations.

Following ChIP DNA quantification via the Quant-iT PicoGreen assay, 1.5 ng of DNA was obtained via diluting ChIP DNA in the appropriate amount of dH_2_O. A single well in a 384-well PCR plate contained a target/condition combination which consisted of 6 μl of diluted DNA (1.5 ng total), 4 μl of diluted primer (both forward and reverse), and 10 μl of 2X SYBR Green PCR Mastermix (Life Technologies). Genes were amplified on a ABI Prism 7900HT Sequence Detection System (Applied Biosystems, Foster City, CA, USA) with a thermal profile consisting of 40 cycles of the following: 50 °C for 2 minutes, 95 °C for 10 minutes, 60 °C for 1 minute, 95 °C for 15 seconds, 60 °C for 15 seconds and finally 95 °C for 15 seconds.

### Continuous Young’s Modulus gradient PEGDM hydrogel fabrication and characterization

Continuous Young’s modulus gradient PEGDM hydrogels were fabricated using this computer controlled, multi-syringe pump device. PEGDM solutions of three different concentrations (5%, 15% and 50%) were dissolved in Opti-MEM media (Life Technologies) containing 0.1% Irgacure 2959 (Ciba Specialty Chemicals, Basel, Switzerland). The computer controlled, multi-syringe system was used to dispense 15% and 50% PEGDM solutions into a custom mold. These solutions were dispensed in an inverse ramp manner, ranging from 55 ml/hr down to 0 ml/hr over 90 seconds. The 5% PEGDM solution was dispensed at a constant rate of 10 ml/hr. The PEGDM hydrogels were subsequently photopolymerized by exposure to 2.3 mJ/cm2 UVA light for a period of 5 minutes, then submerged in Opti-MEM medium for storage. The Young’s moduli were determined using a TA.XTplus texture analyzer (Stable Micro Systems, Surrey, England) by cutting 5 mm gradient sections and analyzing them with a 0.25 inch spherical probe. The probe penetrated the hydrogels at a constant velocity of 0.01 mm/s, and force (newtons), depth (mm), time (s) and strain data were collected, and the Young’s modulus was computed using the following equation (E2):


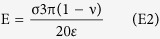


where ν is the poisson ratio and *ε* is the strain of polyethylene glycol^39^.

### Polyurethane acrylate topographic pattern fabrication

Controlled topographical patterns of 16 varying line width and space were fabricated from poly urethane acrylate (PUA) resin (Minuta Technologies, Gyeonggi-Do, South Korea) containing 10% (v/v) acrylic acid. Using a soft-lithography technique, 20 μl of the PUA/acrylic acid mixture was dropped onto a polystyrene slide (Thermo Scientific) and polydimethylsiloxane (PDMS) stamps with defined line and space patterns were subsequently pressed onto the PUA/acrylic acid droplet. This construct was subjected to UV light (3 mW/cm2) for 10 minutes, after which the stamp was removed. The resultant patterned substrate was cured for an additional 6 hours under UV light and then washed with warm 2-propanol (Fisher Scientific) for 3 hours, and then thoroughly rinsed in dH_2_O prior to cell culture.

## Additional Information

**How to cite this article**: Kim, J. J. *et al*. Optical High Content Nanoscopy of Epigenetic Marks Decodes Phenotypic Divergence in Stem Cells. *Sci. Rep.* **7**, 39406; doi: 10.1038/srep39406 (2017).

**Publisher's note:** Springer Nature remains neutral with regard to jurisdictional claims in published maps and institutional affiliations.

## Supplementary Material

Supplementary Information
