# Supplementary material for: Optical High Content Nanoscopy of Epigenetic Marks Decodes Phenotypic Divergence in Stem Cells
Source: Sci Rep. 2017 Jan 4;7:39406. doi: 10.1038/srep39406 (PMC5209743; doi:10.1038/srep39406)
Supplement: Supplementary Information [file srep39406-s1.pdf]

SREP-16-27260-T **Optical High Content Nanoscopy of Epigenetic Marks Decodes Phenotypic Divergence in Stem Cells**

Joseph J. Kim, Neal K. Bennett, Mitchel S. Devita, Sanjay Chahar, Satish Viswanath, Eunjee A. Lee, Giyoung Jung, Paul P. Shao, Erin P. Childers, Shichong Liu, Anthony Kulesa, Benjamin A. Garcia, Matthew L. Becker, Nathaniel S. Hwang, Anant Madabhushi, Michael P. Verzi, and Prabhas V. Moghe

SUPPLEMENTARY INFORMATION

Descriptor	Equation	Intuitive Description	H3K4K27me3 Organization
Entropy	$\sum_{i,j} P_{i,j} \log P_{i,j}$	Measure of randomness of GLCM values<br>High entropy: large variations in GLCM values extracted from an image<br>Low entropy: increasingly homogenous GLCM values	Orderly patterns of H3K4K27me3 that tend to repeat throughout the nuclear space will yield lower entropy values, whereas greater disorder and variation yield higher entropy values.
Energy	$\sum_{i,j} P^2{}_{i,j}$	Measure of homogeneity of GLCM values<br>High energy: increasingly uniform distribution of GLCM values from a ROI<br>Low energy: increasingly heterogeneous GLCM values	Repeating patterns of pixel values and general uniformity throughout the nucleus yield higher energy values, whereas lack of uniformity yields lower energy values
Inertia	$\sum_i \sum_j (i-j)^2 \left(P_{i,j}\right)$	Measure of variations present in local regions of contrast<br>High inertia: higher spatial frequencies corresponding to large differences in a contiguous set of pixels<br>Low inertia: lower spatial frequencies	Greater contrast in small ROIs throughout the nucleus will yield high inertia values, whereas a lack of contrast in such ROIs will yield lower inertia values. Thus, high differences between inertia values of H3K4me3 and H3K27me3 indicate a shift in balance towards one mark over the other and a lack of bivalency, whereas smaller differences reflect increasing bivalency
Inverse Difference Moment	$\sum_{i,j} \frac{P_{i,j}}{1+(i-j)^2}$	Measure of local regions of homogeneity<br>High IDM: Higher presence of locally uniform windows in GLC matrix<br>Low IDM: Higher presence of locally heterogeneous windows in GLC matrix	Similar to energy, IDM is a reflection of the presence or absence of uniformity, but it is sensitive to smaller regions throughout the nucleus. Thus, “clusters” of uniformity of H3K4K27me3 will yield high IDM values, whereas a lack of uniformity in even small ROIs throughout the nucleus will yield low IDM values
Correlation	$\sum_{i,j} \frac{(ij)p(i,j) - \mu_x \mu_y}{\sigma_x \sigma_y}$	Measure of gray-level linear dependency<br>High Correlation: scale of local texture is larger than the distance<br>Low Correlation: scale of local texture is smaller than the distance	Increased presence of linear patterns of H3K4K27me3 yield higher correlation values, whereas lack of image linearity yield lower correlation values
Information Measure of Correlation 1	$\frac{entropy + \sum_i \sum_j p(i,j) \log\{p_x(i) p_y(j)\}}{\max(entro\square y_x, entropy_y)}$	Measure of gray level linear dependency with respect to directional entropy. Provides a more "natural" measure of correlation due to its invariance under logarithmic transformation<br>High IMC1: Greater presence of heterogeneous GLCM values in linear directions<br>Low IMC1: Uniformity of GLCM values in linear directions	Lack of linearity of H3K4K27me3 expression yields higher values of IMC1, whereas repeating patterns in linear directions yield low values.
Information Measure of Correlation 2	$\sqrt{1 - \exp\left[-2.0\left[-\sum_i \sum_j P_x(i) P_y(j) \log\{P_x(i) P_y(j)\}\right]\right]}$	Measure of gray level linear dependency with respect to randomness of spatial dependency.<br>High IMC1: Higher presence of repeating patterns in linear directions<br>Low IMC2: Lack of uniformity or repeating patterns in linear directions	Consistent patterns or overall uniformity of H3K4K27me3 expression in local linear directions yield higher IMC2 values, whereas low IMC2 values indicate lack of local linearity
Sum Average		Measure of GLCM distribution relationship to mean intensity accumulations<br>High sum average: indicative of higher presence of punctate regions of high intensity	The accumulation of H3K4me3 and/or H3K27me3 to smaller ROIs leads to the appearance of brighter "spots" studded throughout the nucleus, which yields higher sum average values. This can

	$\sum_{i=2}^{2N_g} iP_{x+y}(i)$	Low sum average: lack of presence of such punctate regions	also be an indication of increased bivalency. Low sum average values indicate a lack of such "spots" in more uniform H3K4K27me3 distribution, or a lack of accumulation to common areas, which can be reflective of the overall absence of bivalency.
Sum Entropy	$-\sum_{i=2}^{2N_g} P_{x+y}(i)\log\{P_{x+y}(i)\}$	Measure of GLCM relationship to distribution of intensity with respect to entropy<br>High and low values correspond similarly to entropy values	Orderly patterns of H3K4K27me3 that tend to repeat throughout the nuclear space yield lower sum entropy values, whereas greater disorder and variation yield higher sum entropy values.
Sum Variance	$\sum_{i=2}^{2N_g} (i - \text{Sum Entropy})^2 P_{x+y}(i)$	Measure of GLCM relationship to distribution of intensity with respect to variance<br>High sum variance: greater standard deviation of sum average<br>Low sum variance: low standard deviation of sum average	Greater variation of H3K4K27me3 accumulated spot size yields higher sum variance values, whereas similar accumulated spot sizes and less variation yield lower sum variance values.
Difference Average	$\sum_{i=0}^{N_{g-1}} iP_{x-y}(i)$	Measure of GLCM relationship to mean intensity differences	Increased bivalency yields lower difference average values, whereas a lack of bivalency yields higher difference average values.
Difference Entropy	$\sum_{i=0}^{N_{g-1}} P_{x-y}(i)\log\{P_{x-y}(i)\}$	Measure of GLCM relationship to intensity differences with respect to entropy	Increased disorder/variation in H3K4K27me3 expression yield lower difference entropy values, whereas increased uniformity/repeating patterns yield higher difference entropy values.
Difference Variance	$\sum_{i=0}^{N_{g-1}} (i - \text{Sum Entropy})^2 P_{x-y}(i)$	Measure of GLCM relationship to intensity differences with respect to variance	Uniform distribution of spot sizes yield higher difference variance values, whereas a wider distribution yield lower difference variance values.

Supplementary Table 1: Mathematical definitions of each Haralick texture descriptor, with accompanying intuitive description and potential insight regarding H3K4K27me3 organization.
$P(i,j)$ = (i,j)th entry in the normalized gray-tone spatial dependence matrix. $P_x(i)$ = ith entry in marginal-probability matrix obtained by summing the rows of P(i,j). N_g = number of distinct gray levels in quantized image. μ_x & μ_x = means of P_x & P_y .
σ_x & σ_y = standard deviations of P_x & P_y. P_{x+y} = probability of co-occurrence matrix coordinates summing to $x+y$.

Gene	Primer Sequence
OCT4	Fwd: 5'-GAGCAGAAGGATTGCTTTGG-3'<br>Rev: 5'-AAAACCGGGAGACACAACTG-3'
NANOG	Fwd: 5'-AAAGTTTTATCCCATTCCTG-3'<br>Rev: 5'-TTAATCCCGTCTACCAGTCT-3'
LIN28	Fwd: 5'-TGTTTCTGATTGGCCAGCGC-3'<br>Rev: 5'-GTCTCTGACACCTCTGGGGT-3'
PAX6	Fwd: 5'-CCTTCACTTGACCGCTCAAG-3'<br>Rev: 5'-ACCCACTAATCACTCCGCAACA-3'
TUBB3	Fwd: 5'-CCCTTCCGAGCTCTGATCC-3'<br>Rev: 5'-CTGAGCTTTTGCCGGTTTT-3'
NESTIN	Fwd: 5'-TACCTCTCTCGGATGTGTTG-3'<br>Rev: 5'-AGCGACTGAGAGTCGGGAGTG-3'
BGLAP	Fwd: 5'-CGC TCTCAGGGGCAGACACT-3'<br>Rev: 5'-GCACCCTCCAGCATCCAGTA-3'
RUNX2	Fwd: 5'-CCACCCGGCCGAACTGGTCC -3'<br>Rev: 5'-CCTCGTCCGCTCCGGCCCACA -3'
PPAR-γ	Fwd: 5'-TATTCAGCCTGCAGTCTCATTTGG-3'<br>Rev: 5'-CCAGAGTCCGGAGTTTTGGAGTT-3'
LPL	Fwd: 5'-GAGATTTCTCTGTATGGCACC -3'<br>Rev: 5'-CTGCAAATGAGACACTTTCTC -3'

Supplementary Table 2: Forward and Reverse primer sequences targeting the promoter regions of POU5F1, NANOG, NESTIN, PAX6, BMP2, RUNX2, PPARG, RHOA used in Chromatin Immunoprecipitation assays.

Examples of Dominant Texture Descriptor Groups	Microenvironments: "Epigenetic" Constraints		
	**Chemical Growth Factors**	**Mechanical Stiffness**	**Nanotopography**
H3K4me3 Uniformity	+++	+++	++
H3K27me3 Uniformity	+++	+++	++
Bivalency Uniformity	++	+++	++
Correlational Metrics	++	+	++++

Supplementary Table 3. Approximate distribution of relative influence of descriptor groups based on 10 "+" signs per condition. "Uniformity" refers to values of entropy, energy, inertia, inverse difference moment. "Correlational metrics" refer to correlation, info measure of correlation 1, info measure of correlation 2. "Bivalency" is concerned with sum and difference values.

Remarks: Qualitative observations of differentiating cells reveal that the intensity of H3K27me3 increases as cells commit to a particular lineage (**Figure 2**). However, intensity based descriptors of H3K4K27me3 were not sufficient for distinguishing differently differentiated phenotypes. hMSCs cultured on the nanogrooved substrate exhibit a clear shift in intensity balance from H3K4me3 to H3K27me3 as line/space patterns widen, however intensity features alone were not sufficient in accurately classifying topographies that were several hundred nanometers in width difference from each other (**Supplementary Figure 17**). Another feature that was explored was the size, shape and number of nucleoli in differentiating cells. However, upon analysis of these features, it became clear that they were not sufficient for distinguishing different cell types from each other (**Supplementary Figure 11**). This is most likely due to the fact that all cells analyzed were cell-cycle synchronized, and it has been reported that nucleoli patterns and growth dynamics change according to where cells are along in their cell cycle [59]. Thus, texture-based descriptors of epi-mark organization proved to be uniquely adept at detecting intranuclear pattern changes in response to specific microenvironmental cues, where specific aspects of texture respond differently to different cues (i.e. chemical, mechanical, topographical), as outlined above (**Supplementary Table 3**).

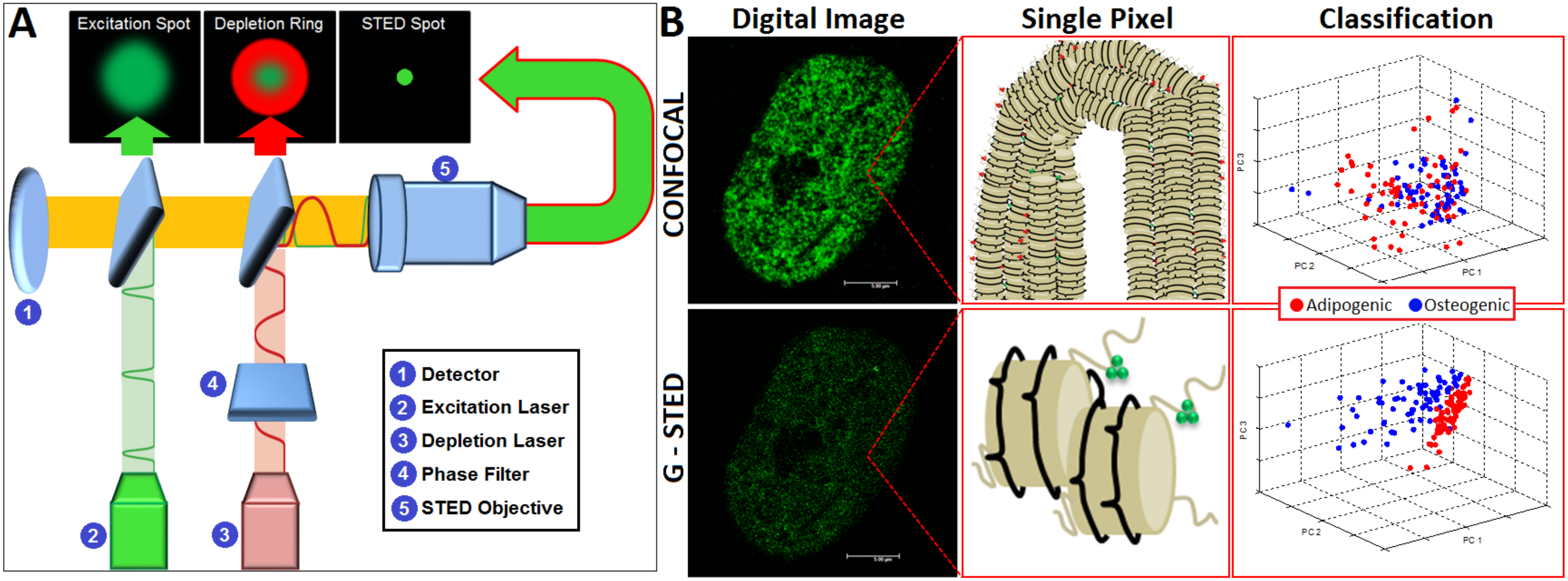

Supplementary Figure 1: Super-resolution nanoscopy is necessary to resolve distinctions in Epi-mark organization. (A) Schematic of Stimulated Emission Depletion (STED) Nanoscopy. The optical diffraction limit of approximately 200 nm is crossed via depleting an outer ring of fluorescence based on point spread function, to ultimately achieve a resolution under 50 nm. (B) Comparison of conventional confocal and time-gated STED images of hMSC nuclei labeled with H3K4me3. Scale bar = 5 um. Schematics of nucleoli / chromatin configurations able to be captured in single pixels of conventional confocal and STED images. A 200 nm resolution limit with (D) conventional confocal limits single pixels to capturing the expression of over 20 nucleosomes in a solenoid, looping chromatin configuration, whereas a sub 50 nm resolution ability with (E) STED enables single pixels to capture 1-5 nucleosome units. Classification of texture descriptors extracted from confocal versus G-STED images of adipogenic and osteogenic hMSCs labeled for H3K4K27me3 demonstrate the necessity of G-STED resolution for accurate classification.

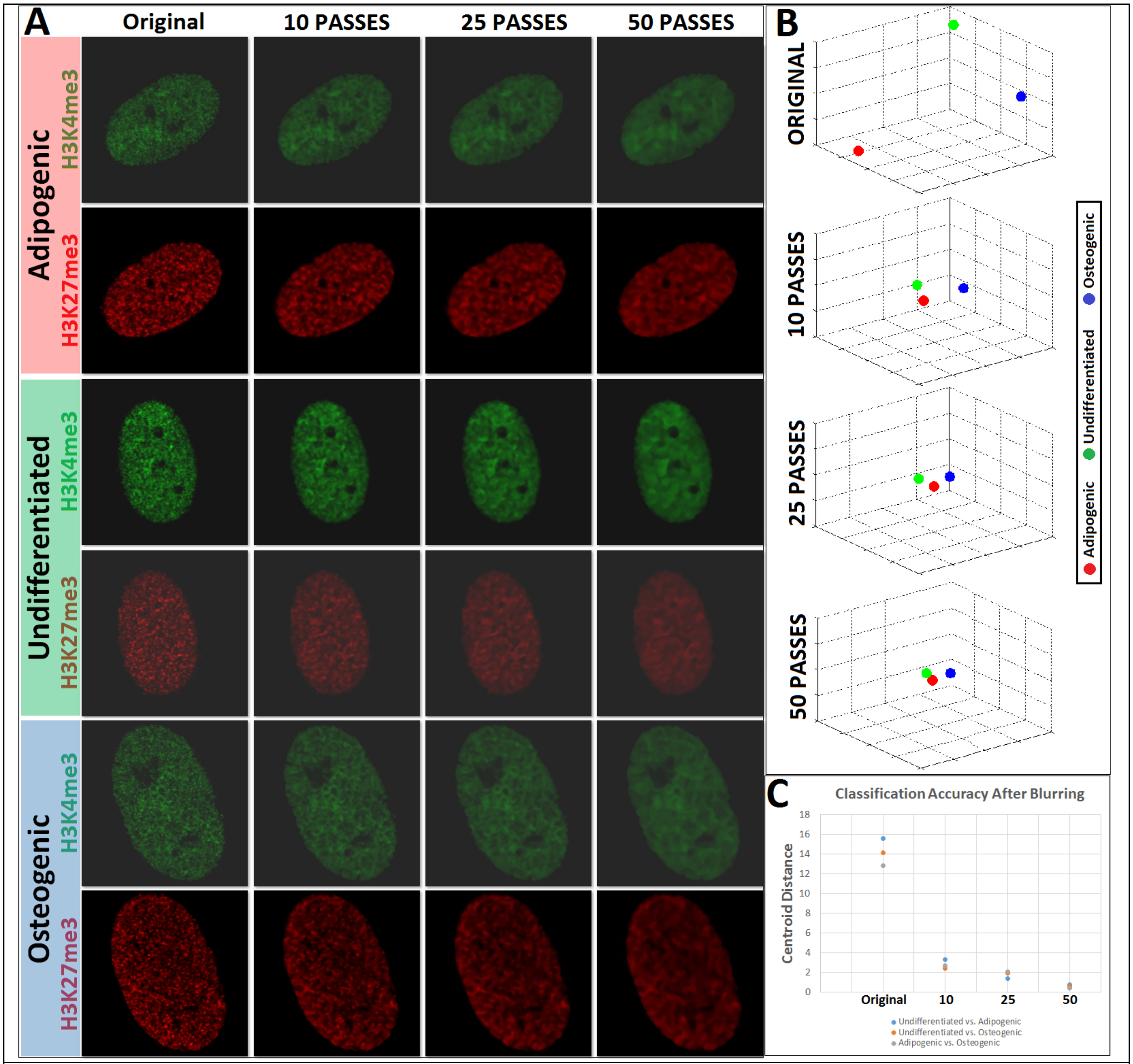

Supplementary Figure 2: Classification accuracy of texture descriptors of H3K4K27me3 labeled cell types decreases with increased blurring.(A) A 7x7 median filter, which replaces every pixel with the median value of a surrounding 7x7 sliding window, was applied with 10, 25 and 50 passes, resulting in an increasing blurring effect with increasing passes. (B) Descriptors of blurred and the original unblurred image were plotted onto PCA plots, revealing a decrease in classification accuracy with increased passes of the median filter. (C) Distances between centroids were computed, as an indication of classification accuracy, and reveal that distances sharply decrease after application of the median filter, and continue to decrease gradually with increasing passes.

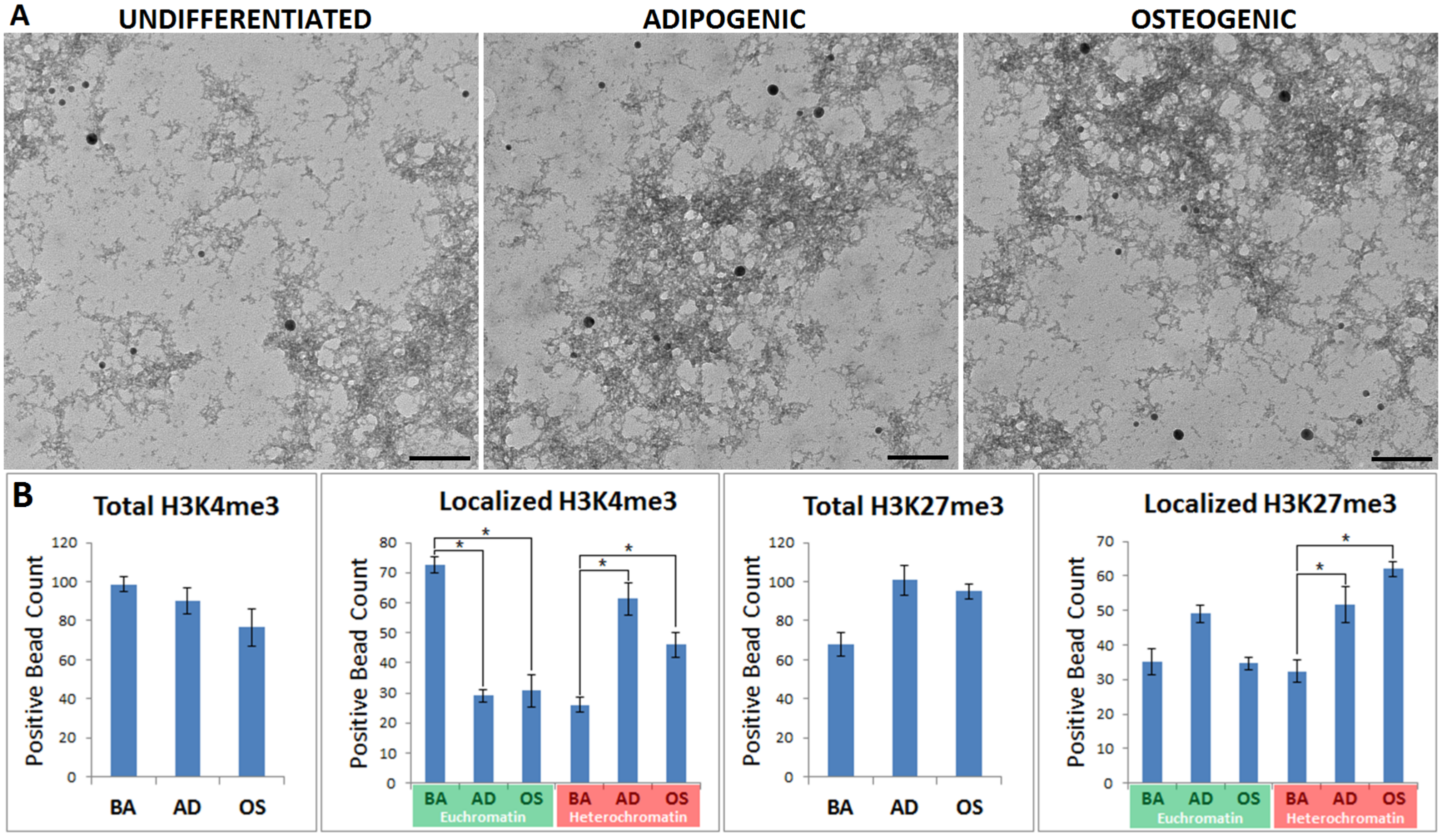

Supplementary Figure 3: Immunoelectron micrograph quantification of H3K4me3 and H3K27me3 marks reveals distinct distribution of both marks in euchromatic and heterochromatic areas. (A) Representative TEM micrographs of the isolated nuclei of undifferentiated hMSCs and hMSCs exposed to adipogenic cues, osteogenic cues, which were immunolabeled with colloidal gold beads (K4me3: 6 nm, K27me3: 10 nm) and contrast stained with uranyl acetate followed by lead citrate Heterochromatic areas appear dark, whereas euchromatic areas are lighter. Scale bar = 100 nm. (B) Colloidal gold labeled marks were counted from approximately 30 micrographs per condition, from three different hMSC donor lines. The presence of the gene activating mark, H3K4me3, is highest in undifferentiated hMSCs in euchromatin and lowest in heterochromatin, whereas the gene silencing mark, H3K27me3, is relatively more prevalent in both post-mitotic phenotypes in heterochromatin. No statistical differences between groups in total counts (i.e. total H3K4me3 and H3K27me3) were found by one-way ANOVA (K4: $F(2,6) = 2.461936$, $p=0.165701$; K27: $F(2,6) = 8.177932$, $p=0.069332$). When organized by location (i.e. localized H3K4me3 and H3K27me3), statistical differences were determined using one-way ANOVA followed by Tukey's post-hoc analysis. * $p<0.05$.

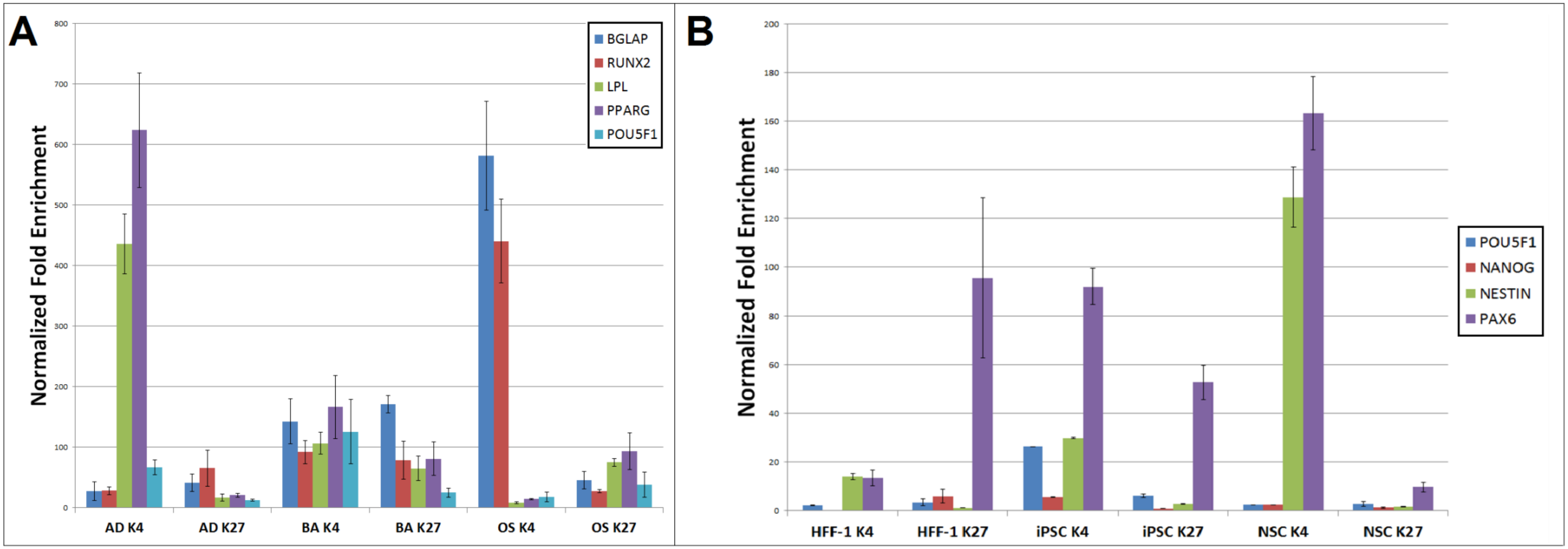

Supplementary Figure 4: Chromatin Immunoprecipitation analysis of H3K4me3 and H3K27me3 in (A) differentiating hMSCs and (B) differentiating iPSCs derived from HFF-1 cells. The activating mark, H3K4me3 (K4), was associated with significantly higher levels of LPL & PPARG in adipogenically induced hMSCs , BGLAP & RUNX2 in osteogenically induced hMSCs , and NESTIN & PAX6 in iPSC derived NSCs . The silencing mark, H3K27me3 (K27), was associated with relatively higher levels of genes that were not expected to be expressed in each respective cell type, and lowest for genes that were. In undifferentiated cells, both H3K4me3 and H3K27me3 are present across most genes, confirming the theory that their co-localization is highest in naïve stem cells with open chromatin structures that are ready to undergo a wider range of lineage specification.

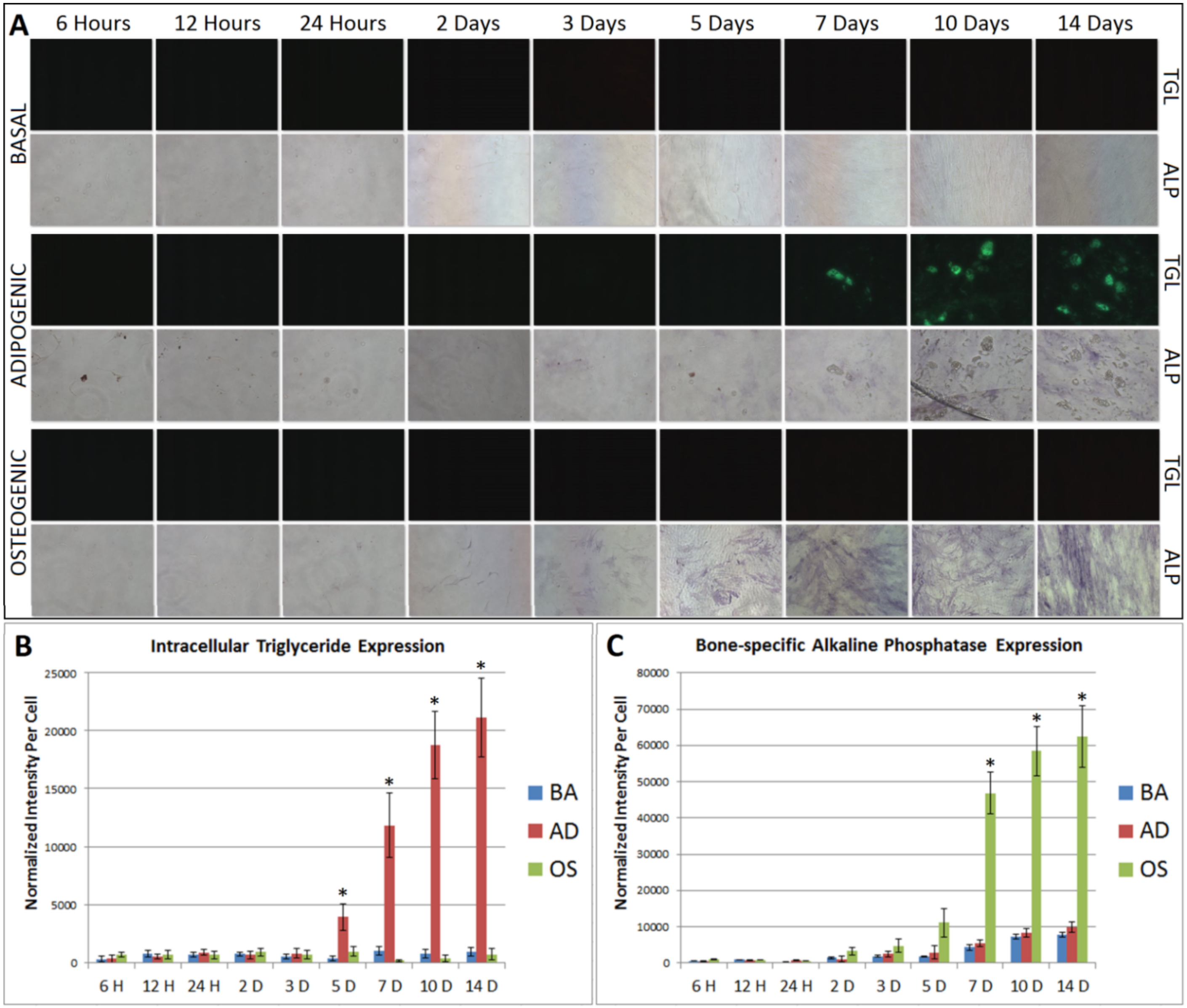

Supplementary Figure 5: Lineage staining quantification of developing hMSCs. (A) hMSCs were induced to differentiate towards adipogenic and osteogenic lineages and degree of differentiation was assessed via staining for intracellular triglyceride (TGL) accumulation and bone-specific alkaline phosphate (ALP) expression, respectively. All images were taken at 10x magnification. (B&C) Quantification of lineage marker stains was made via density/integrity measurements,

normalized to cell count. Distinguishable expression of these markers occurred after 7 days for ALP (B) and 5 days for TGL (C), with steady increased expression thereafter. Statistical significance was evaluated by One-way ANOVA with Tukey’s post hoc test. $*p<0.05$

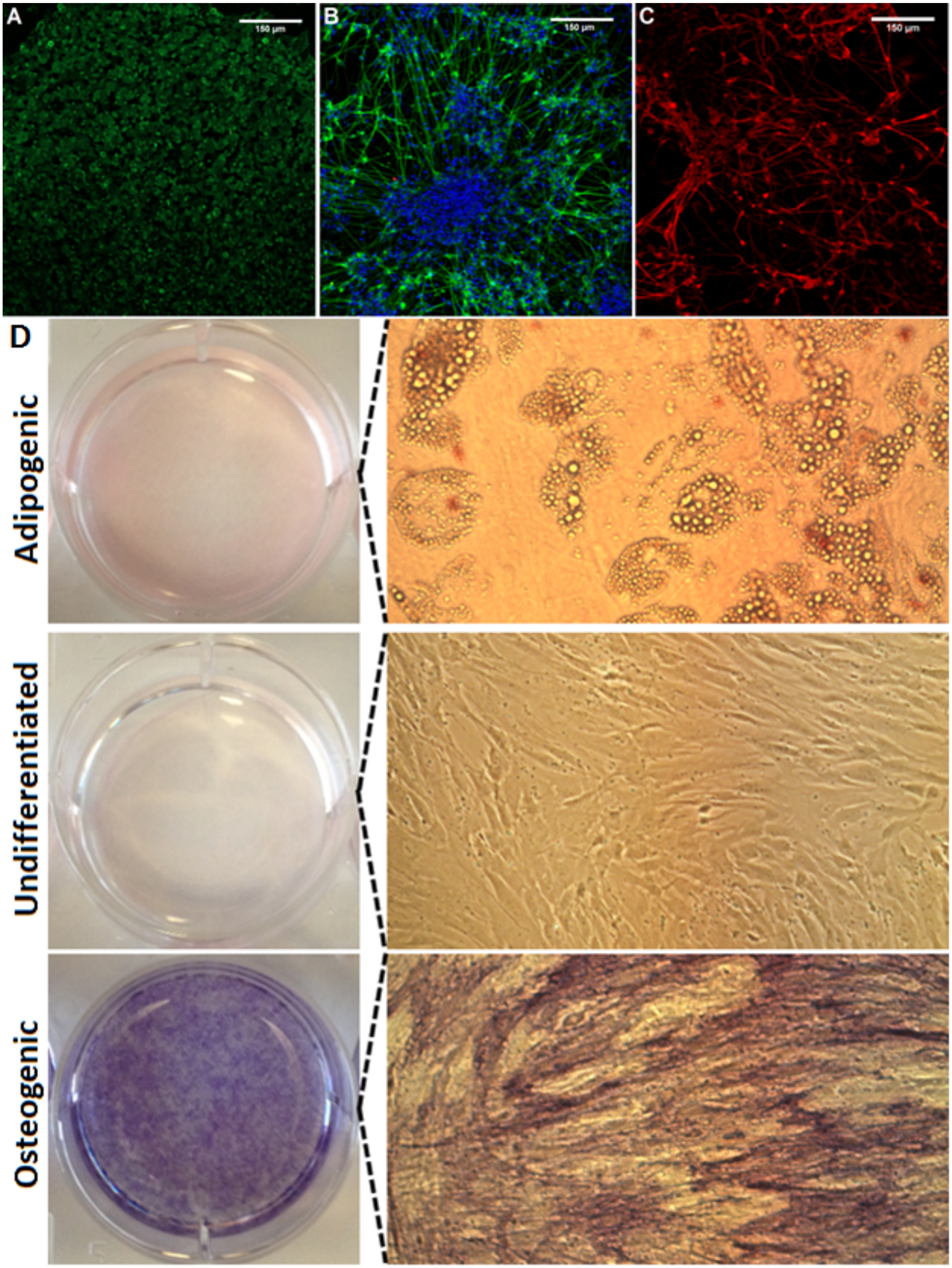

Supplementary Figure 6: Biomarker verification of different cellular phenotypes. (A) iPSCs exhibit robust expression of SSEA4 in green, (B) NSCs express NESTIN in green and their nuclei were labeled with DAPI, and (C) iNs express TUJ1 in red. All images taken at 10x magnification. Scale bar = 150 μm. (D) Differentiated cell types derived from hMSCs were

confirmed with histological staining. Adipogenic hMSCs displayed intracellular triglyceride formation, osteogenic hMSCs stained positive for bone-specific alkaline phosphatase, and undifferentiated hMSCs displayed neither mark.

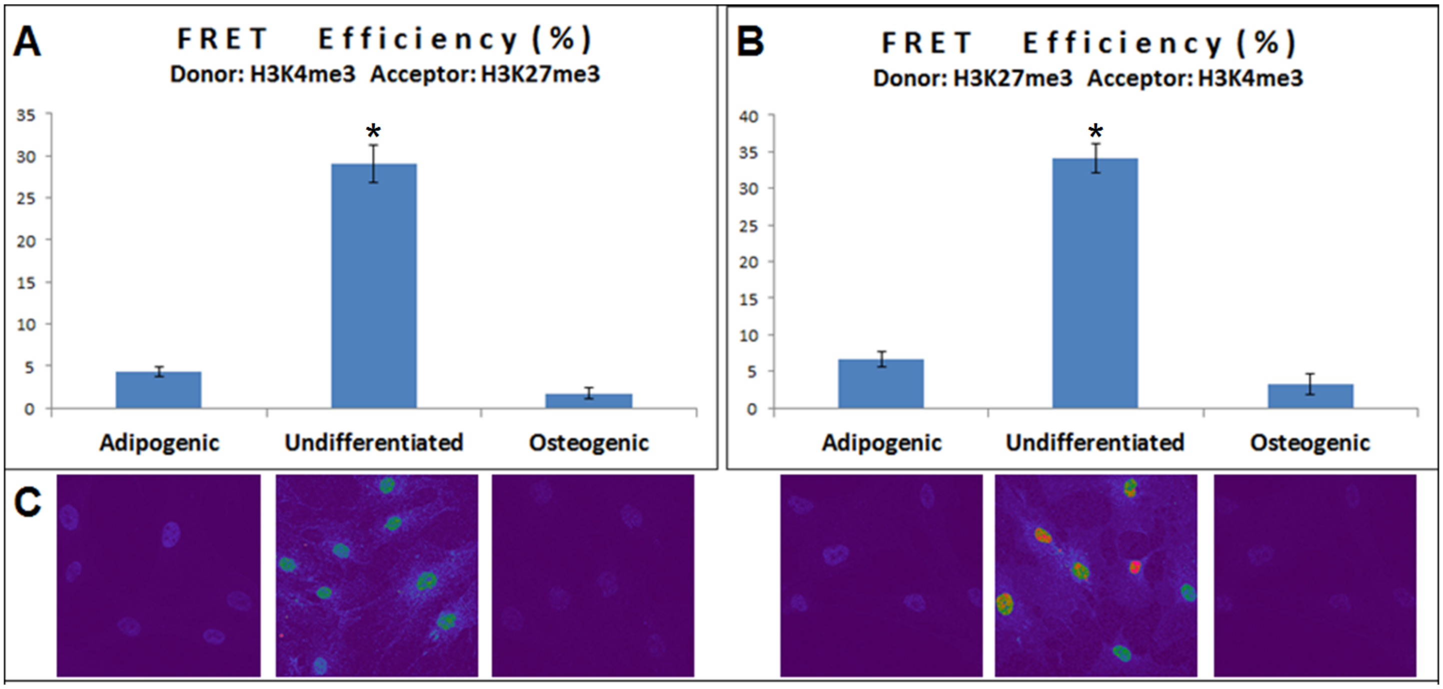

Supplementary Figure 7: FRET Efficiency between H3K4me3 and H3K27me3 reveals increased bivalency in undifferentiated hMSCs. Regardless of designation of donor/acceptor (A or B), the highest FRET signal was found in undifferentiated hMSCs , and significantly less in either differentiated cell type (A&B). Quick look up table (QLUT) heat mapped images further confirm detection of higher FRET signals in undifferentiated hMSCs (C). Statistical significance was evaluated by one-way ANOVA ($F(2,6) = 140.6823$, $p=0.0000091$) followed by Tukey's post hoc test. * $p<0.05$

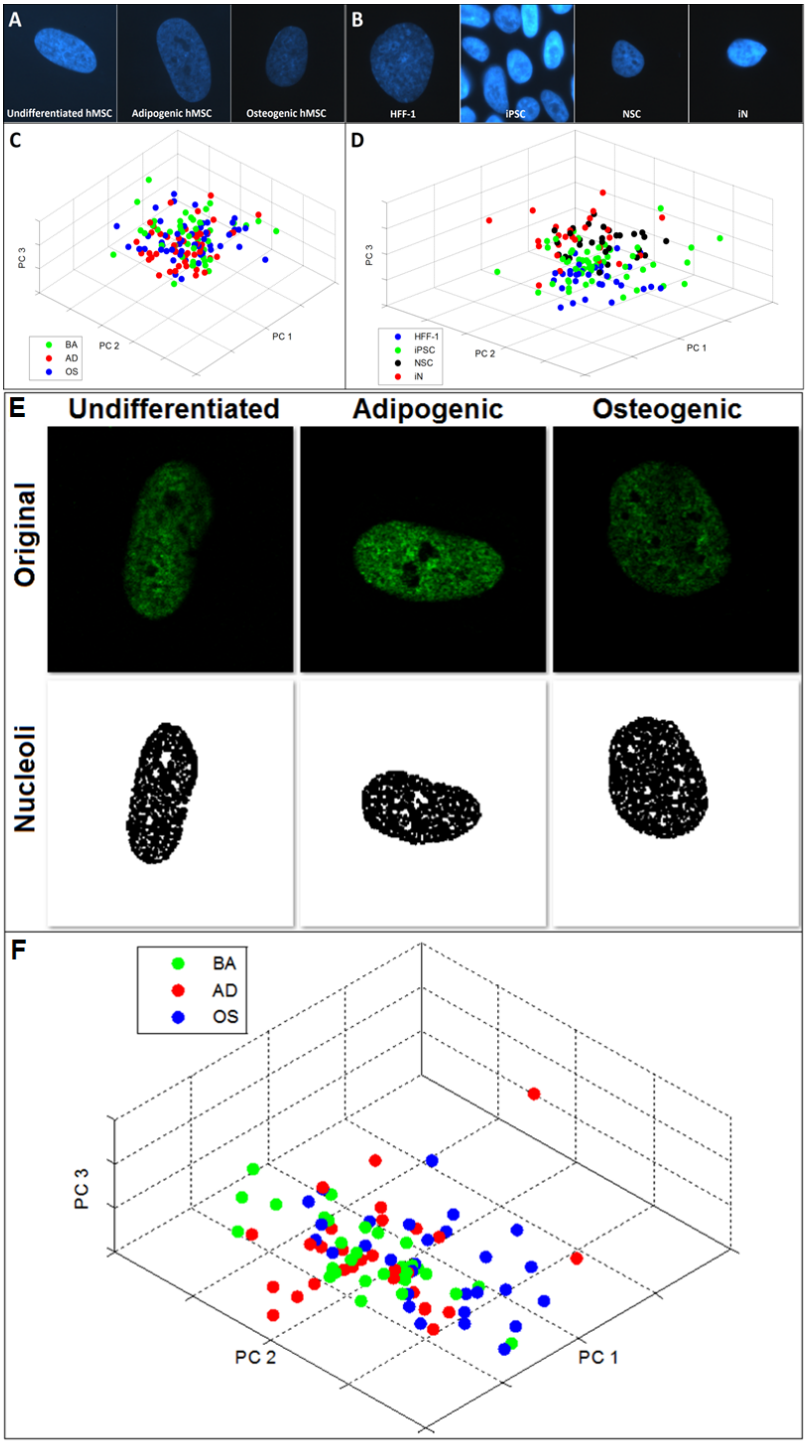

Supplementary Figure 8: High-content image texture analysis of differentiating hMSCs (A) and HFF-1 derived iPSCs (B) labeled with Hoescht reveals the inability to accurately classify different cell phenotypes after PCA analysis (C&D) due to a lack of distinct Hoescht expression in response to different gene transcription programs. (E) Descriptors characterizing geometrical features of nucleoli were obtained by edge detection algorithms (F) However, PCA analysis of these descriptors reveals the inability to accurately distinguish different cell phenotypes based on nucleoli shape, size and number.

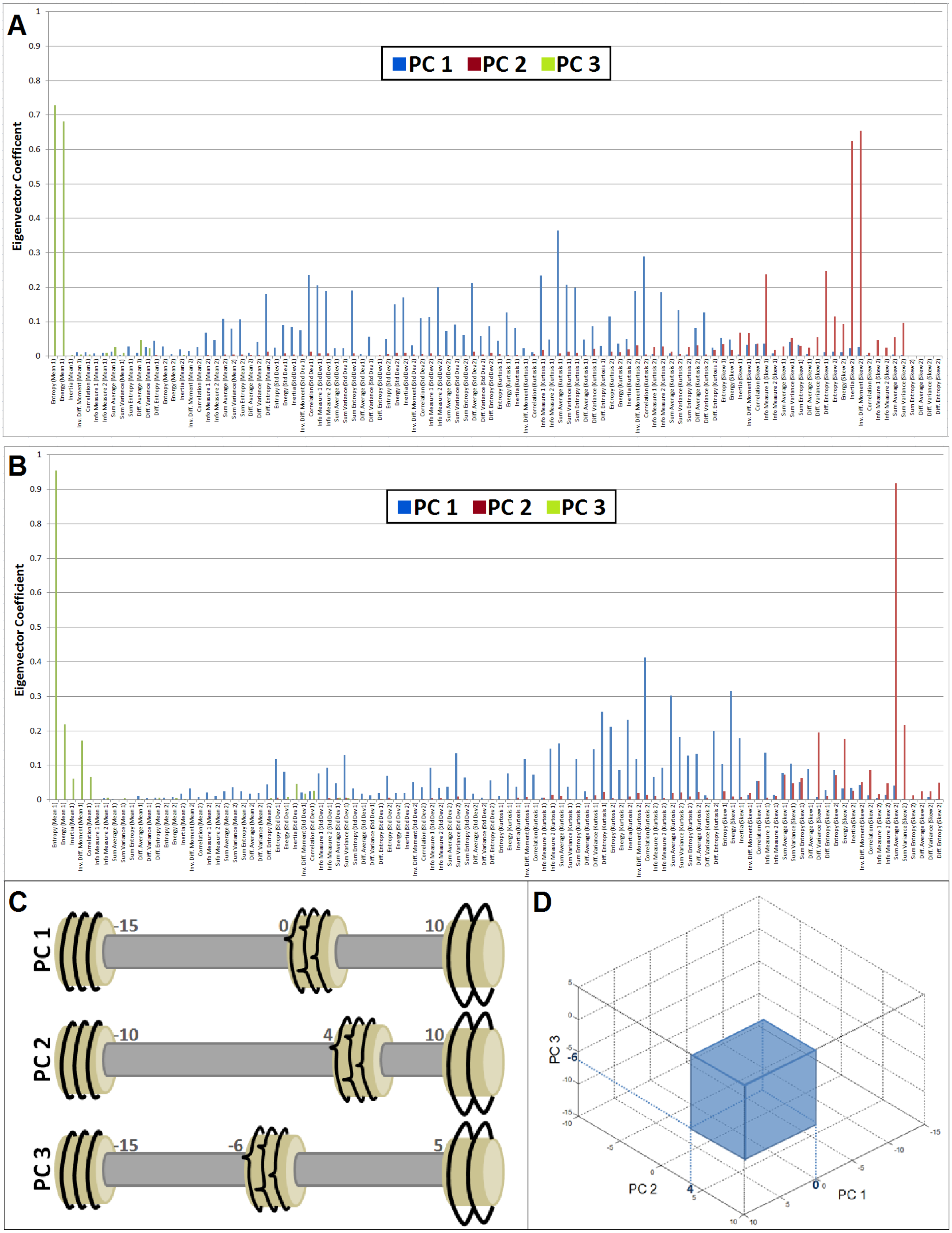
A
Eigenvector Coefficent
PC 1
PC 2
PC 3
B
Eigenvector Coefficient
PC 1
PC 2
PC 3
C
PC 1
-15
0
10
PC 2
-10
4
10
PC 3
-15
-6
5
D
PC 3
-6
PC 2
4
0
PC 1

Supplementary Figure 9: Parsing Index Zone of undifferentiated stem cells in PCA plots. The most influential textural descriptors governing the epimark parsing were probed. The eigenvector coefficients comprising each principal component were plotted for the PCA plots of developing hMSCs (A) and iPSCs (B), with many similarities between both systems. The proximity of undifferentiated hMSCs and iPSCs enabled the establishment of a parsing index zone, occupying a space between 0 and 10 on principal component 1, 4 and 10 on principal component 2, and -6 and 5 on principal component 3 (C). This can be visually represented as a 10 x 6 x 11 three-dimensional cuboid in a PCA plot (D). Examples of influential descriptors are tabulated in **Supplementary Table 3. Remarks regarding the roles of various principal component descriptors are discussed below.**

Remarks: The classification accuracy achieved via PCA analysis indicates the descriptors that display the highest eigenvector coefficients constituting each principal component (PC) (**Supplementary Figure 12A**). **The first PC** is mostly influenced by descriptors quantifying different textures of the summed values of both epi-marks. These values are most sensitive to areas / patterns reflective of bivalency, or the lack of bivalency. For classification, these types of patterns should theoretically aid in distinguishing undifferentiated stem cells versus their differentiated progeny. The specific texture values that display high eigenvector coefficients are **sum average, correlation** and **energy**. **Sum average** may indicate local areas of accumulation of bivalent areas, where higher values correspond to the increased presence of punctate regions of high intensity. This may be insightful for distinguishing a stem versus differentiated state, as the overall abundance of sparse, euchromatic regions in undifferentiated stem cells may be reflected in the lack of presence of these punctate regions of bivalency, whereas the increased presence of dense, heterochromatic regions upon differentiation may be reflected in an increase in these punctate regions, and thus an increase in the value of this descriptor. **Correlation** measures linear dependency patterns in nuclei, with high values indicating that the scale of local texture is larger than the distance, and the opposite for low values. It is well established that heterochromatic regions tend to aggregate along the perimeter of a nucleus along the nuclear membrane [41, 42], thus this correlation descriptor may be detecting increased linear patterns of epi-marks forming along the nuclear perimeter as a stem cell differentiates. The **energy** descriptor reports on levels of homogeneity, thus differences in the general order / disorder ratio of spatial organization of the epi-marks seems to be correlated with phenotype specification. **The second PC** is most influenced by descriptors characterizing the difference between the two epi-marks (i.e. H3K4me3 – H3K27me3). This is notable as these values are most sensitive to capturing any potential shifts in balance between these two marks, as cells differentiate toward different lineages, or are reprogrammed back to a state of pluripotency. As cells begin to commit towards development to a lineage and lose their overall levels of bivalency, subtle differences in the degree of presence of one mark over the other may serve to help specify different lineages from each other, which these descriptors may be increasingly sensitive to. Finally, **the third principal component** is dominated by the heterogeneous / homogenous nature of the activating mark, H3K4me3, which could correspond to distinct spatial patterns of gene program activations in different phenotypes. Since this mark is mainly associated with transcriptional activation, one may speculate that undifferentiated stem cells may have a relatively disorderly spatial organization of H3K4me3 with increased levels of bivalency, and then as cells differentiate, specific spatial areas corresponding to similar gene transcription program activation may be marked, thus resulting in a decrease in the value of entropy and an increase in the value of energy.

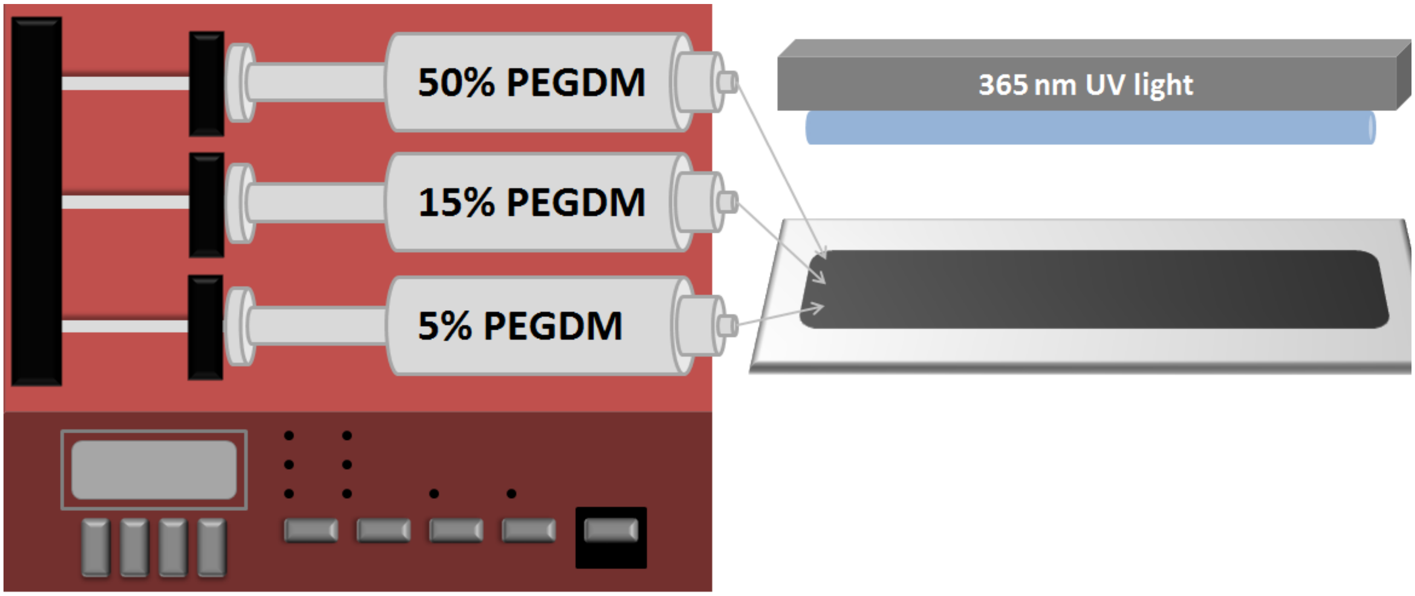

Supplementary Figure 10: Schematic of computer controlled syringe pump device to create continuous Young's modulus gradient PEGDM hydrogels. Different concentration PEGDM solutions were dispensed at different rates into a custom mold of 1 mm depth. PEGDM solutions were subsequently photopolymerized using 2.3 mJ/cm^2 UVA light for 5 minutes.

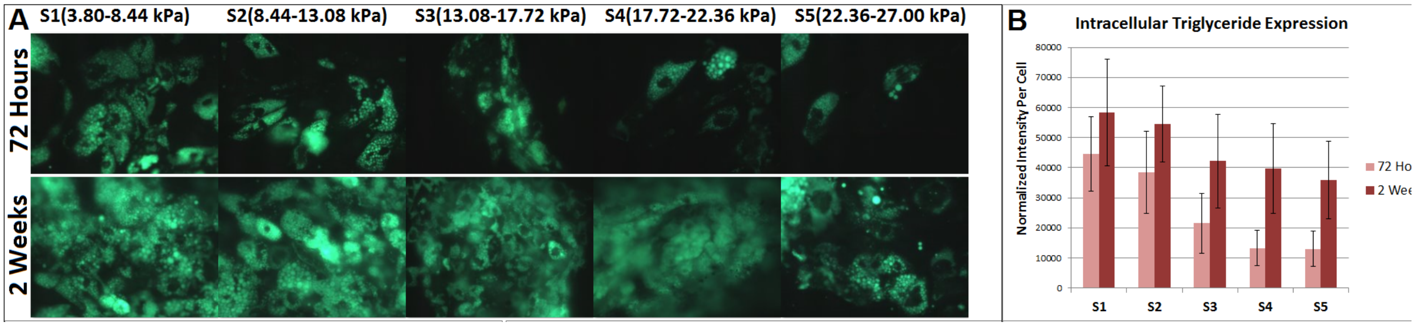

Supplementary Figure 11: Lineage staining quantification of hMSCs cultured on PEGDM continuous Young's modulus gradient hydrogels in MX media reveals increasing TGL expression per cell with decreasing Young's modulus and longer culture time. ALP expression remained relatively low regardless of stiffness.

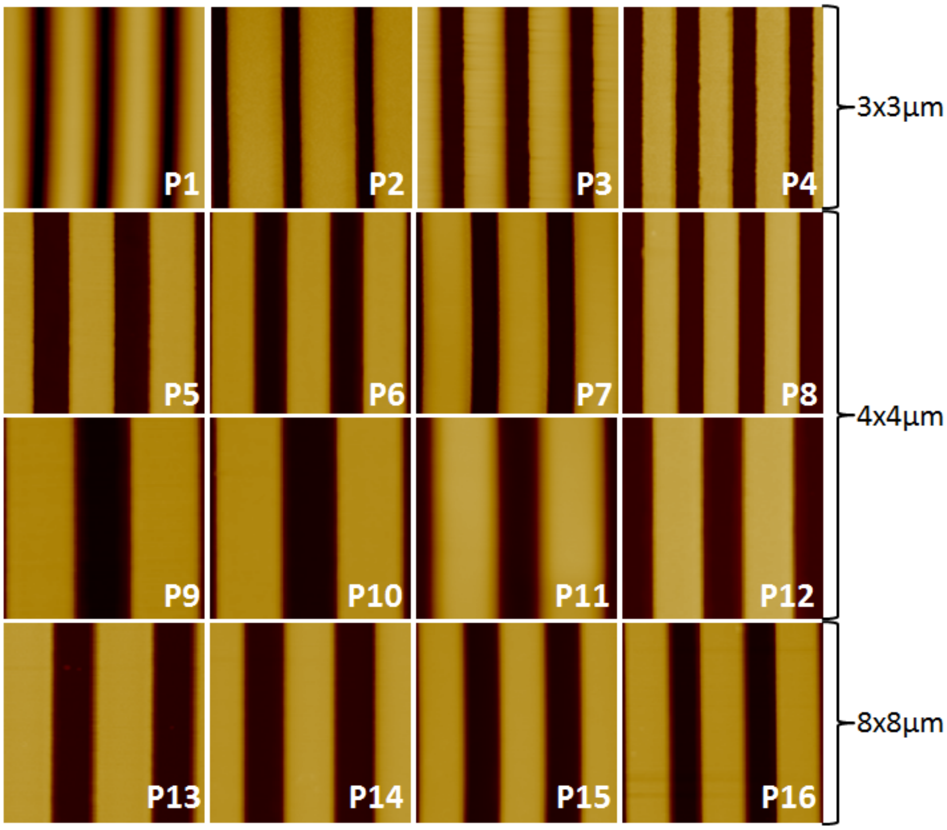

P1	w: 500<br>p: 1000	P9	w: 1250<br>p: 2500
P2	w: 450<br>p: 900	P10	w: 1200<br>p: 2400
P3	w: 400<br>p: 800	P11	w: 1000<br>p: 2000
P4	w: 350<br>p: 700	P12	w: 900<br>p: 1800
P5	w: 800<br>p: 1600	P13	w: 2000<br>p: 4000
P6	w: 750<br>p: 1500	P14	w: 1800<br>p: 3600
P7	w: 700<br>p: 1400	P15	w: 1600<br>p: 3200
P8	w: 600<br>P: 1200	P16	w: 1500<br>P: 3000

w (width), p (period) in nm

Supplementary Figure 12: Atomic Force Microscopy images of PUA topographic patterned substrate. The width of lines/spaces increases from right to left, top to bottom, beginning with the upper right position (P4). The height of the nanogrooves is constant across all patterns at 200 nm.

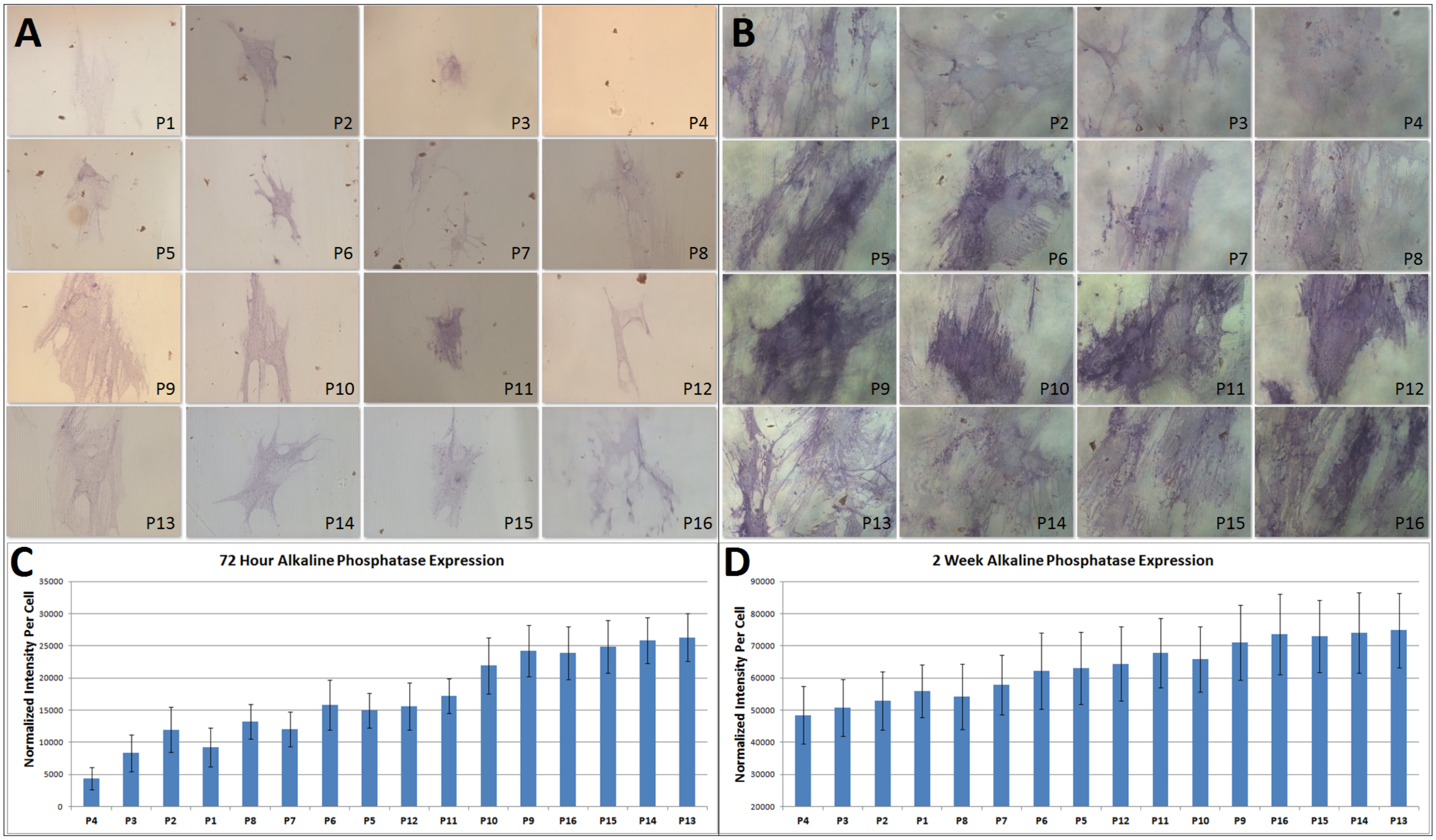

Supplementary Figure 13: Lineage staining quantification of hMSCs cultured on PUA topographic patterned substrate for (A) 72 hours and (B) 2 weeks in MX media. hMSCs were subsequently stained for both TGL and ALP with minimal evidence of TGL staining and increased ALP with increasing line/space width, at both the early (C) and late (D) time points.

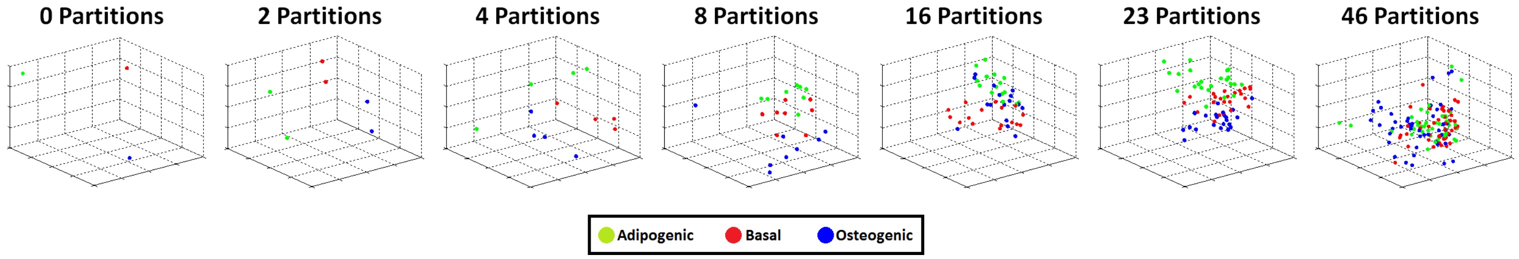

Supplementary Figure 14: Role of ROI partitioning on efficacy of parsing of cell states**.** We investigated how the size of the region of interest (ROI) of nuclear epi-mark texture informatics influenced the overall parsing efficacy between single cells across different lineage fates. One representative nucleus from each of the three phenotypes (i.e. undifferentiated, adipogenic and osteogenic) was chosen randomly, and partitioned into 1, 2, 4, 8, 16, 23 and 46 partitions. The rationale for 23 and 46 is based on the theoretical number of chromosome territories of human nuclei. Our preliminary data indicates that better classification results from a lower number of partitions. That is, increasing the number of partitions tends create more degenerate descriptor data, in terms of distinguishing different phenotypes from each other, although there are "pockets" of partitions that classify well against other "pockets" from different phenotypes. This suggests that unique spatial distribution/organization of these two epi-marks in different phenotypes are manifested on a "global," whole-nucleus scale, whereas when we begin to look at different arbitrary partitions, the data becomes increasingly noisy. Another way to consider this is that these two epi-marks may not be evenly or homogenously manifested in different territories, but rather more distinctly (and phenotype-specifically) distributed in specific chromosomes, and less so in others, which could explain the "partial" classification ability with increased partitions, as well as support the notion that unique patterns of phenotype-specific spatial organization of these marks are most detectable on the whole-nucleus scale.
